# Synergy between the anthocyanin and RDR6/SGS3/DCL4 siRNA pathways expose hidden features of *Arabidopsis* carbon metabolism

**DOI:** 10.1038/s41467-020-16289-3

**Published:** 2020-05-15

**Authors:** Nan Jiang, Aimer Gutierrez-Diaz, Eric Mukundi, Yun Sun Lee, Blake C. Meyers, Marisa S. Otegui, Erich Grotewold

**Affiliations:** 1 Department of Biochemistry and Molecular Biology, Michigan State University, East Lansing, MI 48824 USA; 2 Donald Danforth Plant Science Center, St. Louis, MO 63132 USA; 3 Division of Plant Sciences, University of Missouri, Columbia, MO 65201 USA; 4 Department of Botany, Laboratory of Molecular and Cell Biology, University of Wisconsin-Madison, Wisconsin-Madison, WI 53706 USA

**Keywords:** Metabolic engineering, Epigenetics, Plant genetics, Secondary metabolism

## Abstract

Anthocyanin pigments furnish a powerful visual output of the stress and metabolic status of *Arabidopsis thaliana* plants. Essential for pigment accumulation is TRANSPARENT TESTA19 (TT19), a glutathione *S*-transferase proposed to bind and stabilize anthocyanins, participating in their vacuolar sequestration, a function conserved across the flowering plants. Here, we report the identification of genetic suppressors that result in anthocyanin accumulation in the absence of TT19. We show that mutations in *RDR6*, *SGS3*, or *DCL4* suppress the anthocyanin defect of *tt19* by pushing carbon towards flavonoid biosynthesis. This effect is not unique to *tt19* and extends to at least one other anthocyanin pathway gene mutant. This synergy between mutations in components of the RDR6-SGS3-DCL4 siRNA system and the flavonoid pathway reveals genetic/epigenetic mechanisms regulating metabolic fluxes.

## Abstract (web summary)

*TRANSPARENT TESTA19* (*TT19*) encodes a glutathione *S*-transferase which functions in anthocyanin stabilization and vacuolar transport. Here, by *tt19* suppressor screening, the authors show that RDR6/SGS3/DCL4 siRNA pathway constituents synergistically interact with components of the flavonoid pathway to control carbon metabolism.

## Introduction

The conspicuous colors and non-essential nature of anthocyanin pigments have made their biosynthesis one of the best-studied plant metabolic pathways^1^. Anthocyanins are flavonoid pigments synthesized on the cytoplasmic surface of the endoplasmic reticulum^2^ and transported to vacuoles, where the acidic pH bestow upon them characteristic red, purple or bluish coloration. Flavonoids are derived from primary metabolism, through the general phenylpropanoid pathway, from the amino acid phenylalanine or tyrosine^3^. Anthocyanin pathway enzymes can be divided into those involved in making the anthocyanidin core, and those mediating modifications, including glycosylation, acylation, and prenylation^4^. The extensive knowledge on anthocyanin biosynthesis enzymes from many plants contrasts with how little is known about the mechanisms by which these pigments are transported from their site of synthesis to the vacuole^5,6^. Key players in this process are a group of conserved glutathione *S*-transferase (GST) proteins and ABC transporters that interact with anthocyanins^7–9^ and are believed to facilitate their transport across the tonoplast^10,11^. The first GST described as associated with anthocyanin transport was maize BRONZE2 (BZ2), initially believed to conjugate glutathione to cyanidin-3-*O*-glucoside (C3G)^12^. Subsequent studies in maize and other plants, however, failed to identify anthocyanin-glutathione conjugates^7^.

In *Arabidopsis*, TRANSPARENT TESTA19 (TT19) is the functional homolog of BZ2 and is required for the accumulation of anthocyanins as well as proanthocyanidin (PA) pigments in the seed coat^13^. TT19 shows a broad subcellular distribution and can bind cyanidin as well as C3G^8^. There are eight *tt19* mutant alleles that have been characterized to some degree (Supplementary Table 1). How TT19 and related GST proteins function to promote pigment accumulation remains unclear, but some possible roles for these GSTs include the stabilization of anthocyanins and/or PA precursors, or cooperation with transporters for vacuolar uptake^5^.

*TT19* is controlled by the R2R3-MYB transcription factor PRODUCTION OF ANTHOCYANIN1 (PAP1/MYB75)^14,15^, and to a lesser extent, PAP2/MYB90, MYB113 and MYB114^16,17^. *PAP1* expression, and therefore anthocyanin accumulation, can be induced by high light, likely through the HY5 pathway^18,19^; by high-sucrose levels^16^, such as those found during senescence^20^; or in *Arabidopsis* seedlings grown under anthocyanin inducing conditions (AIC), such as 3% sucrose and strong light^21–23^. *PAP1* mRNA accumulation is also under the control of *TRANS-ACTING SiRNA GENE 4 (TAS4)*^24^, which is cleaved by miRNA828 producing a set of small interfering RNAs (tasiRNAs) in a process that requires RNA-DEPENDENT RNA POLYMERASE 6 (RDR6), SUPPRESSOR OF GENE SILENCING 3 (SGS3), and DICER-LIKE 4 (DCL4)^25,26^. The resulting tasiRNAs include TAS4-siRNA81(-), which targets the mRNAs of *PAP1*, *PAP2*, and *MYB113*^24,27^.

To investigate possible connections between anthocyanin pathway regulation, metabolic flux, and product sequestration, we screened for genetic suppressors that allow anthocyanins to accumulate in seedlings in the absence of TT19 function. Here, we show that all six *tt19-8* suppressors identified correspond to components of the RDR6-SGS3-DCL4 small RNA system. We demonstrate that *PAP1* over-expression is insufficient to restore anthocyanin accumulation in the *tt19* mutant. Indeed, our metabolic analyses show that, contrary to a pulling effect of PAP1 through an increased demand for carbon from the flavonoid pathway, the combination of *tt19* and mutations in *rdr6*, *sgs3* or *dcl4* results in a significant push into the pathway, with three to five times more carbon devoted to flavonoid production. In seedlings, this carbon is derived from sucrose through central metabolism, and from the down-regulation of other metabolic pathways. By combining *rdr6* with *tds4* (impairing the expression of leucoanthocyanidin dioxygenase)^28^, we demonstrate that the increased carbon influx to the flavonoid pathway is not unique to the combination of *tt19* and *rdr6* mutations, but more likely a generalized consequence of the inability to properly accumulate flavonoids. Our findings support a model in which the small RNAs derived from the RDR6-SGS3-DCL4 system provide a regulatory layer over central metabolism that is only exposed when combined with specialized metabolism dysfunctions.

## Results

### Identifying *tt19* suppressor mutations

**Fig. 1:**
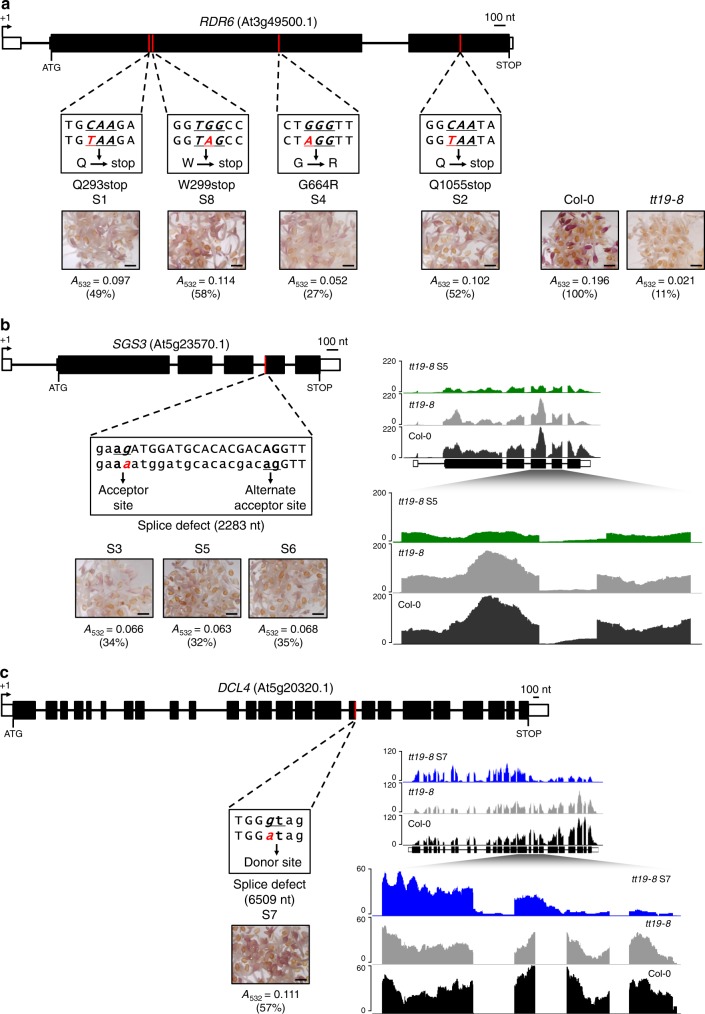
Molecular characterization of the *tt19-8* phenotypic suppressors S1-S8 and corresponding anthocyanin accumulation. **a** Structure of the *RDR6* gene indicating the position and nature of the EMS-induced mutations for the S1, S8, S4, and S2 suppressors. The images show 4-day-old seedlings grown in AIC and accumulating various levels of anthocyanins evaluated by absorbance at 532 nm (A_532_), and normalized to the dry weight of the seedlings. The levels present in Col-0 were taken as 100%. **b** Structure of the *SGS3* gene indicating the mutations for the S3, S5, and S6 suppressors. All three suppressors showed similar levels of anthocyanins in AIC and the same mutation, which abolishes a splicing acceptor site. The genome browser image on the top right shows the reads obtained in RNA-seq experiments for S5, and the region demonstrating the use of an alternate splicing acceptor site in the S5 mutant is shown expanded on the lower image. **c** Structure of the *DCL4* gene indicating the mutation for the S7 suppressor, which abolishes a splicing donor site, as evidenced by the genome browser images comparing the RNA-seq reads of Col-0, *tt19-8*, and S7. Similar anthocyanin quantification results were obtained from at least three independently repeated experiments. Bar, 400 μm.

To investigate the role of TT19 in flavonoid/anthocyanin stabilization and to simultaneously discover components of a possible degradation pathway, we embarked on identifying *tt19* genetic suppressors by mutagenizing homozygous *tt19-8* (SALK_105779C) seeds with ethyl methanesulfonate (EMS). We germinated the M2 seed pools and scored for restoration of anthocyanin pigmentation in 4 day-old seedlings grown under AIC. We identified eight pigmented seedlings and named them *tt19* suppressors S1–S8 (Fig. 1 and Supplementary Fig. 1). Based on the anthocyanin content of seedlings grown in AIC, compared to wild-type (100%) and *tt19-8* (11%), the eight *tt19* suppressors were grouped as strong (49–58%) (S1, S2, S7, and S8), or weak (27–35%) *tt19* suppressors (S3, S4, S5, and S6) (Fig. 1).

The eight *tt19* suppressors were back-crossed with *tt19-8* and F2 seeds (BC_1_F_2_) were grown in AIC. The resulting seedlings showed the expected segregation of ~25% pigmented and ~75% non-pigmented, confirming that the *tt19* suppressing mutations were all recessive. Using bulked segregant analysis by whole-genome re-sequencing, we found that the eight causative mutations were located in two regions. The *tt19* suppressors S1, S2, S4, and S8 corresponded to allelic mutations in At3g49500 (*RDR6*), with the S1 mutation replacing Q with a stop codon at position 293 (RDR6^Q293stop^), S2 replacing Q with a stop codon at position 1,055 (RDR6^Q1055stop^), S4 changing G664 for R (RDR6^G664R^), and S8 changing W to a stop codon at position 299 (RDR6^W299stop^) (Fig. 1a and Supplementary Fig. 2). In S3, S5, and S6, the same G-to-A mutation at nucleotide position 2283 of At5g23570 (*SGS3*) caused a splice defect at the 3’-end of intron 3 in which the normal acceptor site was mutated. The alignment of *SGS3* transcript reads from S5 suggested an alternative splicing site 15 nt downstream of nucleotide 2,283 that resulted in the deletion of five amino acids (Fig. 1b). In S7, a G-to-A change at nucleotide position 6,509 resulted in a splice defect at the 5’-end of intron 17 of At5g20320 (*DCL4*), which was confirmed by RNA-seq (Fig. 1c). In summary, an unbiased screen for phenotypic suppressors of *tt19-8* resulted in all the mutations mapping to genes encoding components of the RDR6-SGS3-DCL4 small RNA system (Table 1).

**Table 1:** Gene mutations underlying the *tt19* suppressors.

Suppressor	Chromosome	Position	Mutation	Reference amino acid	Strand	Accession
S1	3	18352329	G to A	Gln (Q) to Stop	—	At3g49500
S2	3	18349621	G to A	Gln (Q) to Stop	—	At3g49500
S4	3	18351216	C to T	Gly (G) to Arg (R)	—	At3g49500
S8	3	18352310	C to T	Trp (W) to Stop	—	At3g49500
S3/S5/S6	5	7945406	G to A	Splice defect (Donor loss)	+	At5g23570
S7	5	6862758	C to T	Splice defect (Acceptor loss)	—	At5g20320

To confirm that the identified mutations were responsible for suppressing the anthocyanin pigmentation deficiency of *tt19-8*, we crossed *tt19-8* to characterized reference mutants for *RDR6*, *SGS3*, and *DCL4* (*rdr6-11* and *rdr6-15*; *sgs3-11* and *sgs3-14*; and *dcl4-2e* and *dcl4-2t*). We confirmed that homozygous double mutant seedlings for *tt19-8 rdr6*/*sgs3*/*dcl4* accumulated anthocyanins in AIC (upper row, Supplementary Fig. 3). Consistent with *PAP1* being controlled by *TAS4* via the RDR6-SGS3-DCL4 system^24^, the single *rdr6*, *sgs3*, and *dcl4* mutant seedlings accumulated more anthocyanins than the wild-type (Col-0) control, at levels comparable to *PAP1-D* seedlings, in which *PAP1* is under the control of four copies of *CaMV35S* promoter^15^ (lower row, Supplementary Fig. 3). Similar restoration of the anthocyanin pigmentation phenotypes were observed in *tt19* suppressor plants at different developmental stages grown on soil (Supplementary Fig. 4). In contrast to the restoration of anthocyanin accumulation in vegetative organs, none of the eight *tt19* suppressors was able to reinstate the seed coat PA pigmentation characteristic of wild-type and significantly reduced in *tt19-8* plants (Supplementary Fig. 5).

### *PAP1* is insufficient for restoring *tt19* pigmentation

**Fig. 2:**
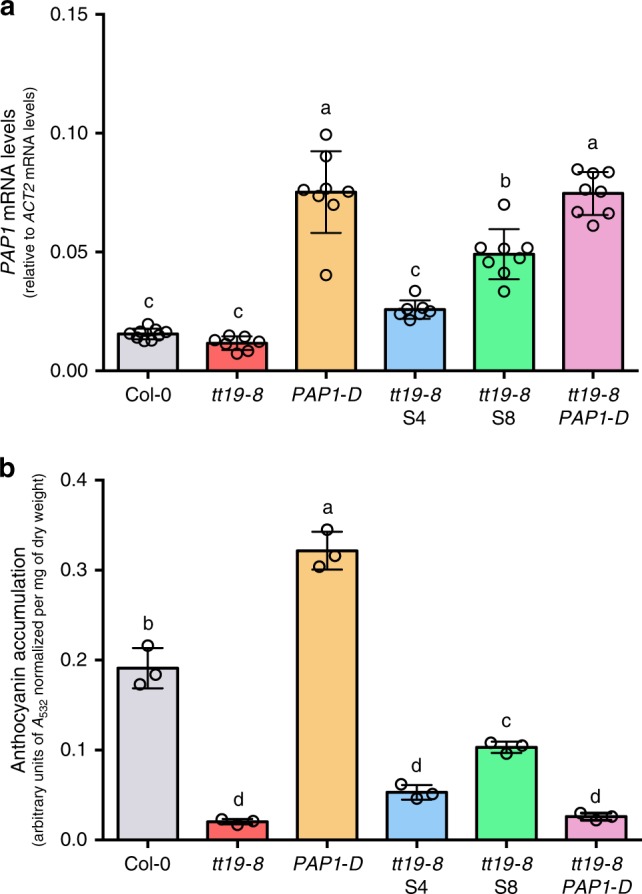
Over-expression of *PAP1* is not sufficient to suppress the anthocyanin deficiency in *tt19-8*. **a** *PAP1* mRNA accumulation levels were evaluated by RT-qPCR and normalized to *ACT2* between the various lines of RNA extracted from 4-day-old seedlings grown in AIC. *n* = 9, 7, 8, 7, 8, 8 biological replicates. **b** Anthocyanin levels from three independent experiments were measured by A_532_ (normalized per mg of dry weight). The error bars represent the standard deviation of the average. Different letters indicate significant differences between the lines based on one-way ANOVA with Tukey’s Honest Significant Difference test (*P* < 0.05). Source data are provided as a Source Data file.

A possible explanation for why mutations in either *RDR6*, *SGS3*, or *DCL4* overcome anthocyanin pigment deficiency in *tt19-8* plants is that more anthocyanins are produced by preventing *PAP1* transcript degradation via *TAS4-siRNA81*^24^ and the subsequent upregulation of anthocyanin biosynthesis genes^15^. To test whether over-expression of *PAP1* alone can overcome the need for TT19, we crossed *tt19-8* to *PAP1-D* plants^15^. *PAP1* mRNA levels were about fivefold higher in *PAP1-D* seedlings in AIC, compared to Col-0 seedlings (Fig. 2a), and *PAP1-D* seedlings showed ~60% increase in anthocyanin accumulation when compared to Col-0 (Fig. 2b). Although the *PAP1* mRNA steady-state level in *tt19-8 PAP1-D* seedlings was comparable to that of *PAP1-D* (Fig. 2a), the *tt19-8 PAP1-D* line failed to accumulate more anthocyanins than the *tt19-8* seedlings (Fig. 2b). The *tt19* suppressor lines that have mutations in *RDR6* resulted in an elevation of *PAP1* mRNA levels and an increase in anthocyanin levels relative to *tt19-8*. However, compared to *tt19-8 PAP1-D* seedlings, the *tt19-8* S4 and S8 lines accumulated larger quantities of anthocyanins, despite lower *PAP1* mRNA levels (Fig. 2). These results demonstrate that, although *PAP1* upregulation is likely necessary for enhanced anthocyanin accumulation in the *tt19* suppressor lines, it is clearly insufficient to overcome the inability of *tt19* to accumulate high anthocyanin levels. Therefore, the mechanisms by which the *tt19* suppressors restore anthocyanin accumulation involve other factor(s), besides *PAP1*.

### Flavonoid accumulation changes in suppressor lines

*Arabidopsis* accumulates at least 15 distinct anthocyanins (A1-A15)^29,30^, most of which are detectable under the metabolic stress conditions imposed by AIC^22,31^. To compare the anthocyanin composition of the suppressor lines with those present in Col-0 and *tt19-8*, we grew homozygous seedlings in AIC and analyzed their anthocyanin profiles. Under AIC, the prevalent anthocyanins in Col-0 were A3, A5, and A7 to A11, with A11 being the most abundant (Supplementary Fig. 6a). Despite *tt19-8* accumulating only ~10% of the anthocyanin content in Col-0, the relative peak abundance remained unaltered (Supplementary Fig. 6b). The anthocyanin profiles of the suppressor lines were also indistinguishable from those of wild-type and *tt19-8*, despite accumulating overall different pigment quantities (Fig. 1; Supplementary Fig. 6b).

**Fig. 3:**
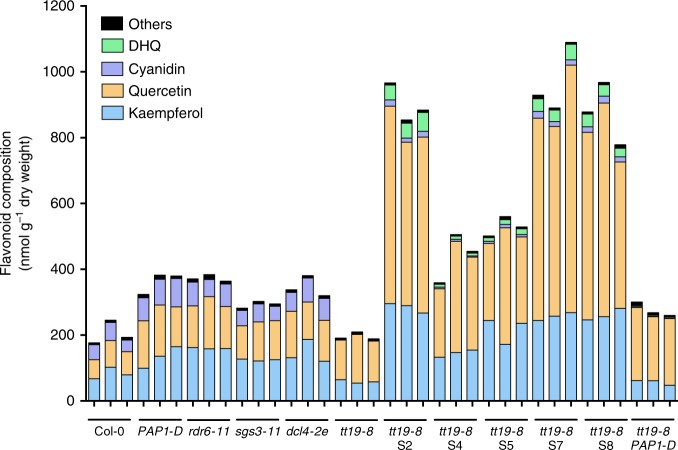
Flavonoid composition of 4-day-old *Arabidopsis* seedlings grown in AIC of the genotypes indicated. Compounds were evaluated by LC-MS/MS against a set of authentic standards of known concentration. Blue, kaempferol; orange, quercetin; purple, cyanidin; green, dihydroquercetin (DHQ); and black, others include apigenin, luteolin, naringenin, eriodictyol, dihydrokaempferol, pelargonidin, epicatechin, and peonidin. Source data are provided as a Source Data file.

As previously reported, *tt19* mutants accumulate significantly larger quantities of flavonols than wild-type plants^8^. Thus, we analyzed the flavonol aglycone composition of seedling from the different genotypes grown in AIC. Our results showed that *tt19-8* seedlings had an ~25% increase in total flavonol content, primarily represented by quercetin, compared to Col-0. Unexpectedly, S2, S4, S5, S7, and S8 showed 4- to 9-fold increases in quercetin and 2- to 3-fold increases in kaempferol compared to Col-0, with S2, S8, and S7 having the highest levels (Fig. 3). To determine whether this increase in flavonol accumulation in the suppressor lines reflected an overall increase in other flavonoid classes, the samples were subjected to acid hydrolysis and then the absolute amounts of the flavones apigenin and luteolin, the flavanones naringenin and eriodictyol, the flavanonols dihydrokaempferol (DHK) and dihydroquercetin (DHQ), the anthocyandins perlargonidin, cyanidin, and peonidin, and the flavan 3-ol epicatechin were measured (Fig. 3 and Supplementary Fig. 7). The most significant differences were observed for DHQ, which is almost undetectable in Col-0 and *tt19-8*, but showed a 20- to 100-fold increase in the suppressor lines, compared to Col-0 (Supplementary Fig. 7). Again, providing evidence that these metabolic changes were independent of the activation of *PAP1*, the *tt19-8 PAP1-D* line showed levels comparable to Col-0 for all these compounds, with the exception of naringenin, which was ~6-fold higher, but still present in much smaller absolute quantities than flavonols (Supplementary Fig. 7). No other pathway intermediate was found to accumulate in *tt19-8* to explain the significantly lower levels of anthocyanins in this mutant. These results demonstrate that the combination of mutations in *TT19* and either *RDR6*, *SGS3*, or *DCL4* results in metabolic changes that are significantly different from those found in the single mutants, suggesting a synergistic cross-talk between cellular processes controlled by the RDR6-SGS3-DCL4 system and those modulated by TT19.

### Enhanced sugar metabolism likely source of extra carbon

Our results show that a significantly larger amount of carbon is devoted to flavonoid biosynthesis in the strong suppressor lines, compared to Col-0 or the single mutants (Fig. 3). To determine the possible source of this extra carbon, we performed untargeted metabolic profiling of the extracts obtained from seedlings corresponding to Col-0, *tt19-8*, and five suppressor lines under AIC, resulting in the identification of ~7,000 altered m/z differentially accumulated features (Supplementary Data 1). Pairwise analyses between *tt19-8* and each suppressor line permitted to identify the contrasting features that were mapped to predictive pathways and displayed as pathway cloud plots (Supplementary Fig. 8a–e). Two compounds, likely corresponding to sucrose ([M + Cl]^−^, 377.0859) and glucose ([M + Cl]^−^, 215.0315), were identified as part of dysregulated sucrose and trehalose degradation pathways with a lesser representation in all five suppressor lines than in *tt19-8*, but not different between Col-0 and *tt19-8* (Supplementary Fig. 8). Authentic standards were used to determine the absolute quantities of sucrose and glucose in all the genotypes (Supplementary Fig. 9a, b). The significant decrease in sucrose and glucose levels in the *tt19* suppressor lines compared to *tt19-8* indicated that more sucrose and glucose was being consumed in the *tt19* suppressors, providing the extra carbon necessary for the enhanced flavonoid production.

To determine to what extent the observed increase in sugar (sucrose and glucose) metabolism was a consequence of the high-sucrose levels in the media, we grew *Arabidopsis* seedlings in water in high-light conditions. Anthocyanin accumulation was still evident, but at about half the levels present in high-sucrose media (Supplementary Fig. 9c). Logically, sucrose levels were considerably lower (>90% reduction) in extracts of water-grown seedlings (Supplementary Fig. 9a). Interestingly, the suppressor lines presented a similar reduction in endogenous sucrose and glucose levels as observed when the seedlings were grown in sucrose-rich media, and this reduction was not observed in the *tt19-8* or *rdr6-11* single mutants (Supplementary Fig. 9a, b). These results indicate that the additional carbon required for the increased flavonoid biosynthesis in the suppressor lines could be provided by sugar catabolism.

### Transcript accumulation changes in suppressor lines

To better understand the cross-talk between the RDR6-SGS3-DCL4 system and the TT19 pathway, we carried out RNA-seq on seedlings grown in AIC for Col-0, *tt19-8*, and *tt19* suppressor lines. The *tt19-8* mutant showed differences in mRNA accumulation patterns when compared to Col-0, with 2,642 genes displaying statistically significant (FDR < 0.05) differences (Supplementary Data 2). The genes with ~2-fold reduced expression included those involved in late anthocyanin biosynthesis, consistent with the lower accumulation of anthocyanins in *tt19-8*. However, this reduction in gene expression does not explain why *tt19-8* shows only ~10% of the anthocyanin content of wild-type plants (Fig. 1a).

**Fig. 4:**
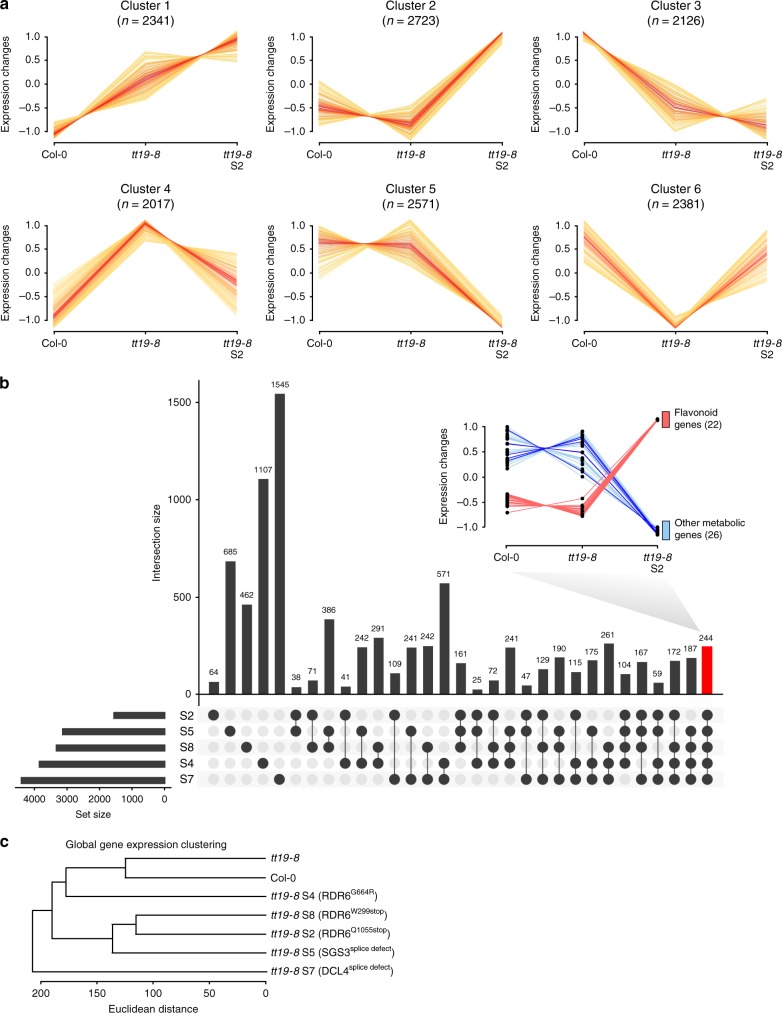
RNA-seq analysis of Col-0 and *Arabidopsis* mutant seedlings grown in AIC. **a** Total differentially expressed genes among Col-0, *tt19-8*, and *tt19-8* S2 were grouped into six clusters. **b** The UpSet plot summarizes all intersections of significantly differentially expressed genes between *tt19* suppressors and *tt19-8*, sorted by degree. The combinations of intersections are indicated as dark circles in the matrix and the numbers of differential expressed genes across various exclusive intersections are represented as vertical columns with number of genes on the top. The bars plot on the bottom left displays the set number of total differentially expressed genes in each *tt19* suppressor line against *tt19-8*. The clustering plot between Col-0, *tt19-8*, and *tt19-8* S2 on the top right indicates that among the 244 shared genes (highlighted with red column) across all suppressors, 22 flavonoid-related genes (pink lines, including early pathway gene *4CL3*; core flavonoid pathway genes *CHS*, *CHI*, *F3’H*, *DFR*, and *LDOX*; anthocyanin-modification genes *UGT78D2*, *UGT79B1*, *3AT1*, *3AT2*, *UGT75C1*, *5MAT*, and *SAT*; flavonol biosynthesis genes *BGLU1* and *UGT73B2*; PA biosynthesis genes *AHA10* and *LAC15*; regulatory genes *TT8*, *TTG2*, and *MYBL2* as well as *ABCC2* and *CHIL*) are part of Cluster 2 and 26 other metabolic genes (blue lines) are part of Cluster 5 in **a**. Dark blue lines display expression changes of six genes that are involved in competitive pathways with flavonoid biosynthesis. **c** Global gene expression clustering analysis on Col-0, *tt19-8*, and *tt19* suppressors (S2, S4, S5, S7, and S8).

Gene expression patterns in the *tt19* suppressor lines were rather different from both *tt19-8* and Col-0. Gene ontology enrichment of *K*-means clusters showed that a large number of genes (captured in clusters 1, 2, 3, and 5, Fig. 4a) exhibited different expression in *tt19-8* S2 (strong suppressor), compared to *tt19-8* or Col-0. Cluster 2 included late anthocyanin pathway genes that showed decreased mRNA accumulation in *tt19-8* and early anthocyanin pathway genes (e.g., CHS and *CHI*) that are also required for flavonol accumulation (Fig. 3, Supplementary Data 3).

One of the genes that showed a dramatic increase in mRNA accumulation in the suppressor lines was At1g02940 (*AtGSTF5*) encoding a *phi*-type GST (Supplementary Fig. 10a). The *Arabidopsis* Next-Gen Sequence DB^32^ shows the existence of a single siRNA that potentially targets At*GSTF5* for degradation. AtGSTF5 is a GST similar to TT19 (Supplementary Fig. 10b), and therefore, we surmised that it might compensate for the flavonoid binding function missing in *tt19-8*. Constructs for wild-type *AtGSTF5* CDS (WT) as well as for an optimized version (OPT) harboring synonymous substitutions across the entire CDS that would render it insensitive to siRNAs were transformed into *tt19-8*. We verified expression of both versions of At*GSTF5* by RT-qPCR in the transgenic plants, yet observed no complementation of the anthocyanin deficiency phenotype. To determine whether *AtGSTF5* is necessary for the restoration of anthocyanin synthesis in the *tt19-8* suppressor lines, we designed two guide RNAs to edit target sequences in exons 2 and 4 of *AtGSTF5* by CRISPR/Cas9 and identified six *tt19* suppressor lines with mutated *AtGSTF5* as homozygous, harboring various indels in *AtGSTF5*. However, when we compared the anthocyanin levels between the *tt19-8* suppressors with wild-type or mutated *AtGSTF5*, we did not detect statistically significant differences (Supplementary Fig. 10). This result demonstrates that AtGSTF5 is unlikely to participate in the stabilization/vacuolar sequestration of anthocyanins, yet its enhanced expression in the suppressor lines, compared to both Col-0 and *tt19-8*, suggests a possible role in another cellular function normally carried out by TT19.

Whereas all *tt19* suppressors had similar effects on anthocyanin accumulation, they showed surprisingly different patterns of gene expression (Fig. 4b), indicating that, while they probably function together as part of the RDR6-SGS3-DCL4 system, each one has separate functions that are independent of the others. The *tt19* S4 (RDR6^G664R^) suppressor is potentially interesting, since it showed an effect on mRNA accumulation that was dramatically different from the effects observed for S2 and S8, both harboring nonsense mutations in *RDR6* (Fig. 4c). Only 244 differential expressed genes were shared by all the five suppressor lines analyzed against *tt19-8* (Fig. 4b, Supplementary Data 4). From these 244 genes, 197 show changes in mRNA accumulation with the same trend (up or down). We rationalized that these 197 genes contained those that are most likely responsible for the ability of the suppressor lines to accumulate anthocyanins in the absence of TT19 function.

As anticipated, among these 197 genes, there were 22 upregulated genes related to phenylpropanoid/flavonoid biosynthesis or regulation, and an additional 26 repressed genes associated with various metabolic or regulatory activities (Fig. 4b, Supplementary Data 4). Among those 26 genes, six have been functionally characterized (or predicted) to be involved in competitive pathways with phenylpropanoid/flavonoid biosynthesis, including *CYP79B3* and *MYB34* involved in indole glucosinolate biosynthesis^33,34^; *CYP84A4* involved in arabidopyrone biosynthesis^35^; *CYP90A1*/*CPD* involved in a critical step for brassinosteroid biosynthesis^36^; *UGT71C1* involved in the decoration of salicylic acid^37^; and flavonol-phenylacyltransferase 1 (*FPT1*) likely participating in phenylalanine consumption^38^. Thus, a shared feature of all the suppressor lines is the increased expression of phenylpropanoid/flavonoid-related genes, and decreased accumulation of gene products likely competing for the early precursors of the pathway (e.g., Phe and Tyr).

To explore the possible link between the increased sugar metabolism providing the extra carbon and those genes differentially expressed in *tt19* suppressor lines compared to Col-0 and *tt19-8*, we analyzed GO term enrichment in the six clusters shown in Fig. 4a. We identified 15 genes under the GO term monosaccharide/hexose/glucose metabolic process only in Cluster 2, corresponding to genes highly expressed in *tt19-8* S2 (Supplementary Fig. 11). An analysis of previous published RNA-seq data for *rdr6-11* and Col-0 from *Arabidopsis* shoots^39^ confirmed that 13 of these genes are highly expressed in *tt19-8* S2, but not in *rdr6-11* alone. Thus, the upregulation of these 13 carbohydrate metabolic process-related genes in the *tt19* suppressors provides one likely explanation for the increased carbon flux towards flavonoids in the suppressor lines.

We investigated whether the synergistic effect of the *tt19* and RDR6-SGS3-DCL4 system mutations on increased flux toward the flavonoid pathway involved small RNAs. We performed a global analysis of small RNAs (20 to 24 nt) between Col-0, *tt19-8*, and *tt19-8* S2 and S7 seedlings, all grown in AIC (Supplementary Fig. 12a and Supplementary Data 5). As anticipated, the suppressor mutations abolished the biogenesis of 21 nt tasiRNAs, which was also evidenced by the increased accumulation of the *TAS4* precursor RNA in all the suppressor lines, as well as increased mRNA accumulation of tasiRNA gene targets, including the *TAS4* targets *PAP1* and *PAP2*^24^, and *TAS3* target *ARF4*^40^. We found that levels of five miRNAs were significantly different between Col-0 and *tt19-8* seedlings (Supplementary Fig. 12b). Significantly higher in *tt19-8* than in Col-0 were miR158b, miR167a-5p and miR399c-5p, while miR161.2 and miR164b-5p showed the opposite pattern (Supplementary Fig. 12b). The increased expression of miR158b in *tt19-8* did not revert to wild-type levels in the S2 and S7 suppressors. Similarly, the low accumulation of miR161.2 in *tt19-8* compared to wild-type, was not restored in the suppressor lines, indicating that the accumulation of miR158b and miR161.2 does not involve the RDR6-SGS3-DCL4 system. In contrast, the effect of the *tt19-8* mutation on the accumulation of miR399c-5p, miR167a-5p, and miR164b-5p was abolished in the S2 and S7 suppressor lines. These results suggest that these miRNAs (or their targets) participate in the cross-talk between the flavonoid pathway and the RDR6-SGS3-DCL4 system.

Since RNA-seq analysis identified 13 carbohydrate metabolic process-related genes with higher mRNA steady-state accumulation in *tt19-8* S2, we mapped the reads from the small RNA-seq analysis to these differentially expressed genes. One of the genes, At3g43190 (*SUS4*), encoding *SUCROSE SYNTHASE 4*, showed increased mRNA levels in *tt19-8* S2 (Supplementary Fig. 12c). Our analyses identified a small RNA (known as siR6611^41^ or AtsRR178278^42^) derived from the *PPR* (Pentatricopeptide repeat) gene (At1g62930) whose expression is significantly decreased in the suppressor lines (Supplementary Data 5), and which may target *SUS4* mRNA for cleavage^43^ (Supplementary Figure 12c and Supplementary Data 6). Indeed, the formation of siR6611 required miR161.2 and tasiRNAs derived from *TAS2*^44–46^, providing one mechanism by which the RDR6-SGS3-DCL4 system and the TT19 pathway may converge on the control of sugar metabolism (Supplementary Fig. 12d).

### Second flavonoid mutant similarly affected by *rdr6* mutation

**Fig. 5:**
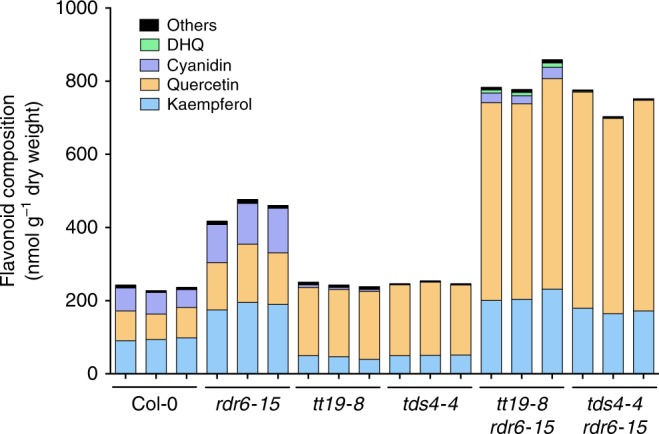
Flavonoid composition of Col-0 and *Arabidopsis* mutant seedlings grown in AIC. Left to right, wild-type Col-0; single mutant *rdr6-15*, *tt19-8*, *tds4-4*; and double mutant *tt19-8 rdr6-15* and *tds4-4 rdr6-15*. Blue, kaempferol; orange, quercetin; purple, cyanidin; green, dihydroquercetin (DHQ); and black, others include apigenin, luteolin, naringenin, eriodictyol, dihydrokaempferol, pelargonidin, epicatechin, and peonidin. Source data are provided as a Source Data file.

The results so far cannot distinguish whether the synergistic effect of *RDR6-SGS3-DCL4* and *tt19-8* on the increase of carbon flux into the flavonoid pathway relates to the absence of TT19 function itself, or to the abnormal flavonoid accumulation that characterizes the *tt19-8* mutant (Fig. 3). To distinguish between these two possibilities, we generated a double mutant between *rdr6-15* and *tds4-4. tds4-4* corresponds to a null allele of the anthocyanidin synthase (ANS) gene, which encodes an enzyme that converts leucoanthocyanidins into 3-hydroxyanthocyanidins^47^, pathway intermediates involved in anthocyanin but not flavonol formation. We then conducted flavonoid aglycone composition analyses in single (*rdr6-15*, *tt19-8*, *tds4-4*) and double (*tt19-8 rdr6-15* and *tds4-4 rdr6-15*) mutant seedlings grown in AIC. The total flavonoid levels of Col-0, *tt19-8* and *tds4-4* were very similar; the main difference being that *tds4-4* seedlings accumulated no anthocyanins (Fig. 5). The total flavonoid levels of *tds4-4 rdr6-15* were comparable to those in *tt19-8 rdr6-15*, and approximately three times larger than those in Col-0 (Fig. 5). These results demonstrate that the synergistic effects observed in the suppressor lines are likely to involve an interaction between components of the RDR6-SGS3-DCL4 system and flavonoid compounds affected in both the *tt19-8* and *tds4-4* mutants.

To further delineate what aspect of flavonoid pathway dysfunction might be responsible for the interaction with the RDR6-SGS3-DCL4 system to increase carbon flux into the pathway, we generated the *tt5-4 rdr6-15* double mutant, in which *tt5-4* corresponds to a mutation in chalcone isomerase, an early pathway step necessary for both flavonol and anthocyanin accumulation (Supplementary Fig. 7). Similar to *tt5-4*, but different from Col-0 or *rdr6-15*, *tt5-4 rdr6-15* seedlings grown in AIC lack the flavonols quercetin and kaempferol, as well as cyanidin, yet show an increase in naringenin chalcone/naringenin (hydrolysis converts naringenin chalcone to naringenin), because of the block in the pathway (Supplementary Fig. 13a–d). When we evaluated the absolute values of glucose and sucrose in the *tt5-4 rdr6-15* double mutant, they were not significantly different from those in Col-0 or single mutants (Supplementary Fig. 13e, f), indicating that it was not the absence of flavonoids that synergized with the RDR6-SGS3-DCL4 system, but an imbalance between different flavonoids caused by the *tt19-8* or *tds4-4* mutants.

We then took advantage of the possibility to feed seedlings grown in AIC with various flavonoids to determine whether the accumulation of flavonols in the absence of anthocyanins would have an effect. For this, we added quercetin or kaempferol, but neither was sufficient to trigger an enhanced catabolism of glucose or sucrose (Supplementary Fig. 13e, f), despite flavonols accumulating at high levels in the seedlings (Supplementary Fig. 13a, b). The addition of naringenin (the product of chalcone isomerase) resulted in flavonol and cyanidin formation in ratios slightly different from those present in Col-0, but still not sufficient to trigger enhanced sugar catabolism (Supplementary Fig. 13). The results suggest that what triggers enhanced sugar catabolism in the presence of a disabled RDR6-SGS3-DCL4 system is the absence of anthocyanins (regardless of whether flavonols are present or not), or a pathway dysfunction, likely characterized by the accumulation of a pathway intermediate specific to the anthocyanin branch of the pathway present in both *tds4-4* and *tt19-8*, but absent in *tt5-4*.

## Discussion

**Fig. 6:**
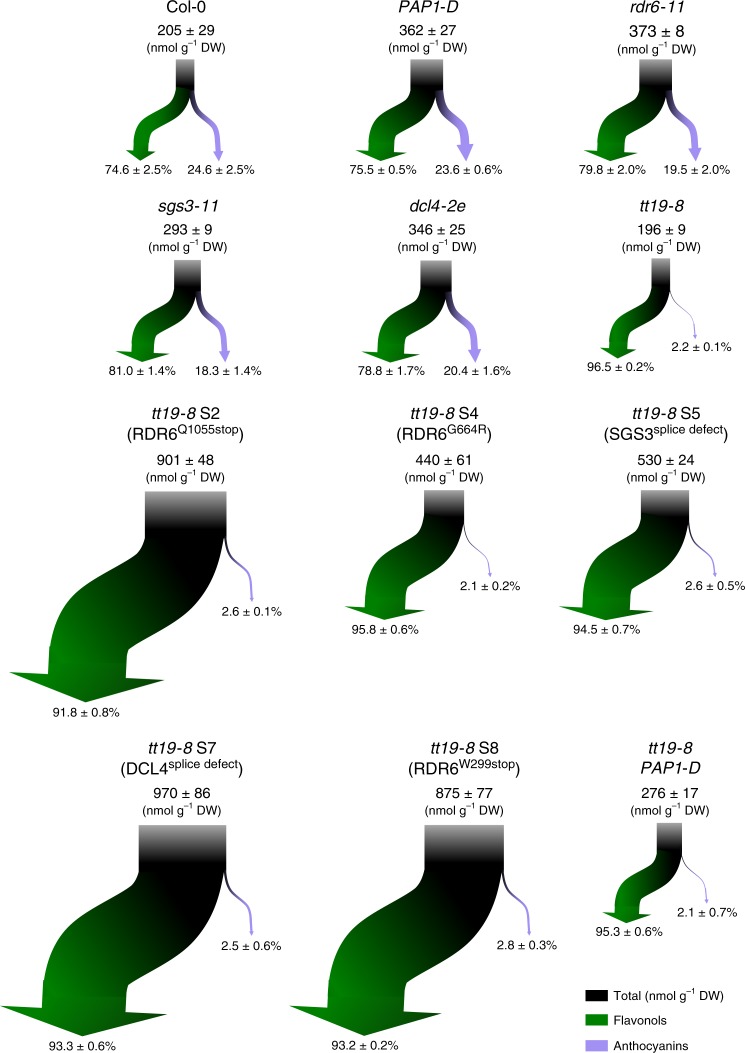
Flavonoid flux maps displaying total carbon input into the different branches of the pathway. The block arrow (black at the top) displays the estimated total input of carbon for flavonoid biosynthesis. Green and purple arrows show the distribution of flavonols and anthocyanins, respectively. The data used for the graphical representation of carbon distribution is provided in Supplementary Fig. 7.

Our quest for phenotypic suppressors of *tt19* resulted exclusively in the identification of multiple recessive alleles of components of the RDR6-SGS3-DCL4 siRNA pathway, which is involved in the post-transcriptional gene silencing of a large number of genes^39^. It is notable that the only identified context in which anthocyanins can still accumulate in a *tt19* mutant is by disabling a major regulatory mechanism that causes global gene expression changes. Our results demonstrate that a consequence of these gene expression changes is a flux increase into the phenylpropanoid/flavonoid pathway, resulting in greater levels of anthocyanins and other flavonoids (Fig. 6). Thus, while our results cannot rule out a role of TT19 in the stabilization, solubilization, and/or vacuolar sequestration of anthocyanins, it is evident that if enough anthocyanins are produced, they can accumulate, even in the absence of TT19. A possibility that we considered is that the gene expression changes promoted by the *tt19* suppressors might induce a TT19-like protein. We identified *AtGSTF5*, which is normally expressed at very low levels in both wild-type and *tt19-8*, as one of such candidates. However, by combining gain- and loss-of-function approaches, we showed that it is neither necessary nor sufficient to overcome the absence of TT19 function (Supplementary Fig. 10). Another possibility that our experiments ruled out is that enhanced expression of *PAP1*, as a consequence of disabling the RDR6-SGS3-DCL4 siRNA system^24,27^, would be sufficient to overcome the need for TT19 (Fig. 2). Indeed, our results show that the augmented anthocyanin accumulation that characterizes *rdr6, sgs3*, and *dcl4* plants^48^ occurs largely through the impaired tasiRNA-mediated degradation of the *PAP1* mRNA. Thus, while increased *PAP1* expression is necessary, it is clearly insufficient to explain the anthocyanin pigmentation of the *tt19* suppressors.

The *tt19* suppressors are characterized by a three to fivefold increase in carbon flow into the flavonoid pathway (Fig. 6). Increase in *PAP1* mRNA accumulation, either by *PAP1-D* or when the RDR6-SGS3-DCL4 siRNA system is disabled (Fig. 6), results in a modest (~1.7 fold) increase in overall carbon to the pathway, likely caused by the transcriptional activation of late pathway genes by *PAP1*^17^, which results in a flavonoid biosynthesis pull. In contrast, we propose that most of the additional carbon dedicated to flavonoid accumulation in the *tt19* suppressor lines is a consequence of carbon being pushed into the pathway, by a synergistic interaction between the loss RDR6-SGS3-DCL4-controlled siRNAs and a flavonoid metabolic dysfunction. In addition, we show that the cross-talk between the impaired RDR6-SGS3-DCL4 system and flavonoid pathway dysfunction is not specific to the loss of *TT19* as a second anthocyanin pathway mutant (*tds4-4*) shows a similar carbon flow synergistic effect with *rdr6* (Fig. 5).

What is the source of the additional carbon pushed into the flavonoid pathway in the *tt19* suppressors? The RNA-seq results demonstrate that part of the carbon is likely coming from pathways that compete with phenylpropanoid/flavonoid biosynthesis (Fig. 4b). However, the results of untargeted metabolic profiling indicated that the majority of the carbon is actually derived from alterations in central metabolism, including carbohydrate degradation (Supplementary Fig. 8). Indeed, sucrose and glucose levels are dramatically decreased in the *tt19* suppressor lines, with little or no changes in the *tt19-8* or *rdr6-11* seedlings (Supplementary Fig. 9). Interestingly, the induction of anthocyanin triggered by *PAP1-D* or *rdr6* had minimal, if any effect on sugar metabolism (Supplementary Fig. 9), indicating that flavonoid increases resulting from pulling can be absorbed by intermediates or by carbon diversion from other pathways, further underscoring the differences between the pull and push mechanisms.

What are possible molecular mechanisms underlying the synergistic interaction between the loss of RDR6-SGS3-DCL4-controlled siRNAs and the flavonoid metabolic dysfunction? The RDR6-SGS3-DCL4 siRNA system controls the expression of a large number of genes^39^, and flavonoids have been shown to play many regulatory roles^49–52^, thus the opportunity for synergistic interactions are multiple, reflected in the rather striking effects on gene expression (Fig. 4). However, the activation in the *tt19* suppressor lines of *SUS4* offers insights on some unexpected molecular mechanisms that could lead to major metabolic alterations. Indeed, miR161.2 accumulation is decreased in *tt19-8*, and does not recover in the suppressor lines (Supplementary Fig. 12b). One of the targets of miR161.2 is a *PPR* gene, which is also targeted by a product of the RDR6-SGS3-DCL4 system on *TAS2*^44,45^. This results in the formation of siR6611, which is predicted to target *SUS4* for degradation^41–43^ (Supplementary Fig. 12c). Our results show that, because of the decreased accumulation of miR161.2 and the disabled RDR6-SGS3-DCL4 system in the *tt19* suppressors, the expression of siR6611 is decreased resulting in *SUS4* mRNA increasing (Supplementary Fig. 12d). Whether miR161.2 or additional miRNAs are affected in other anthocyanin biosynthesis mutants to explain the altered expression of genes involved in central metabolism remains to be determined.

Our findings are consistent with a model in which, under normal conditions, RDR6-SGS3-DCL4-controlled small RNAs maintain the homeostasis of central metabolism. In such conditions, the disabling of the small RNA pathway has modest consequences, reflected in the surprisingly normal phenotype of *rdr6/sgs3/dcl4* mutants^25,53–56^. When a specialized metabolic pathway is perturbed (such as resulting from the *tt19-8* mutation or from *PAP1* overexpression), small RNAs adjust expression of the enzymes and regulators involved in central metabolism to accommodate to this new condition. This model suggests the existence of a surveillance apparatus that communicates metabolic dysfunctions to the RDR6-SGS3-DCL4 system, and miR161.2 could be part of it. However, the many regulatory effects of flavonoids, particularly through the modulation of hormone function^49–52^, and the control of small RNAs by plant hormones (and *vice versa*)^57–59^ provide opportunities for the identification of other components of this surveillance system.

In conclusion, the search for *tt19* phenotypic suppressors resulted in the identification of a cross-talk mechanism between central metabolism and flavonoid biosynthesis that involves the RDR6-SGS3-DCL4 siRNA system. Whether similar mechanisms are involved in the coordination of central metabolism and other specialized metabolic pathway is not yet known, but both small RNAs and specialized metabolites are rapidly evolving^60,61^, providing ample opportunities for emerging synchronization, and for the metabolic engineering of desirable phytochemicals that might be limited by carbon flow from central metabolism.

## Methods

### Chemicals and instruments

Chemical standards (apigenin, luteolin, naringenin, eriodictyol, dihydrokaempferol, dihydroquercetin, kaempferol, quercetin, epicatechin) and HPLC grade solvents were purchased from Sigma (St. Louis, MO). Cyanidin (chloride), pelargonidin (chloride), peonidin (chloride) were purchased from Cayman Chemical (Ann Arbor, MI). Pictures of *Arabidopsis* seeds and seedlings were taken using a dissecting microscope with a Nikon SMZ1500 camera with a Nikon WD54 HR Plan Apo lens. A BioMate 3S UV-visible spectrophotometer (ThermoFisher Scientific, Waltham, MA) was used to measure absorbance.

### Plant materials and growth condition

*Arabidopsis thaliana* (L.) accession Columbia-0 (Col-0), *tt19-8* (SALK_105779), *tds4-4* (SALK_073183), *tt5-4* (GK_176H03), *rdr6-11*, *rdr6-15* (SAIL_617_H07), *sgs3-11*, *sgs3-14* (SALK_001394), *dcl4-2e*, *dcl4-2t* (GK_160G05), and *PAP1-D* lines were obtained from Arabidopsis Biological Resource Center (Columbus, OH). The primers for confirming the indexed T-DNA insertions (listed in Supplementary Table 2) were synthesized by Integrated DNA Technologies (Skokie, Il). All plants were grown in Suremix growth medium (Michigan Grower Products Inc, MI) under controlled conditions (22 °C and 16 h light/8 h dark photoperiod).

### Constructs

For generation of the *AtGSTF5* overexpression lines, two versions of the *AtGSTF5* CDS clones (WT and OPT) were synthesized by ThermoFisher Scientific and subsequently integrated into the pGWB502 binary vector resulting in the *p35S::AtGSTF5* (WT and OPT) constructs via LR reaction. For creation of the At*GSTF5* knockout lines, two CRISPR guide DNAs were designed by CRISPR-Plant (http://www.genome.arizona.edu/crispr/) and cloned into the Cas9 vector pHEE401E via a Golden Gate reaction. The resulting At*GSTF5* overexpression and CRISPR constructs were then introduced into *Agrobacterium tumefaciens* (GV3101) and used for transformation of *tt19-8* and *tt19-8* S2/S7/S8 by floral dip. For the selection of transformed lines, seeds were surface-sterilized and cultured on Murashige and Skoog (MS) solid media including 5% sucrose with hygromycin (50 mg/L).

### Anthocyanin inductive conditions and compound feeding assays

Experiments involving anthocyanin inductive conditions (AIC) were carried out^22,52^. Briefly, surface-sterilized seeds were spread in small Petri dishes with water containing 3% sucrose and incubated two days at 4 °C for stratification. After a cold treatment for two days, the seeds were cultured under continuous cool-white light with 20 xg shaking at 23 °C for four days. For compound feeding assays, *Arabidopsis* seedlings were grown in AIC for three days and then 100 μM of naringenin/quercetin/kaempferol were added for 24 h.

### EMS mutagenesis to isolate *tt19* suppressors

Taking advantage of the very low anthocyanin accumulation of *tt19-8* seedlings, we screened 2.5 g (~125,000) of EMS-treated seeds for potential dominant suppressors by scoring restoration of anthocyanin pigmentation of 4 day-old M_1_ seedlings grown in AIC. We then transferred ~10,000 of the M_1_ seedlings to soil, grew to maturity and collected into ~300 pools of M_2_ seeds. The recessive suppressors that restored anthocyanin accumulation were isolated from the M2 seed pools germinated under AIC.

### Anthocyanin extraction and composition analysis

*Arabidopsis* seedlings were grown in AIC for four days. Seedlings were then lyophilized and dry tissue weighted. Total anthocyanins were extracted with 50% methanol containing 3% formic acid at 200 μg of dry weight per μL of extraction buffer, overnight at room temperature. To evaluate total anthocyanin concentration, the extracts were subjected to spectrophotometry and absorbance at 532 nm was normalized to the dry weight of the tissue (and expressed as A_532_/mg of dry tissue). For HPLC, 20 μL of the acidic methanol extracts were analyzed with a 2695 Alliance HPLC system (Waters Corp., Beverley, MA) on a Symmetry C18 column (Waters, 3.5 μm 4.6 × 75 mm) at 35 °C with flow rate 1 mL/min. Buffer A corresponds to 5% formic acid in water and buffer B to 5% formic acid in acetonitrile. The gradient running condition was: 0 min, 100% A; 20 min, 75% A and 25% B; 22 min, 20% A and 80% B; 22.1 min, 100% B; 25 min, 100% B; 25.1 min, 100% A; and 30 min, 100% A. Anthocyanin profiles were recorded at 532 nm and individual anthocyanin peaks from the HPLC were collected and applied to the LC-MS/MS to determine anthocyanin composition^52^.

### Flavonoid quantification

Aglycone flavonoids were prepared by HCl hydrolysis^62^. Briefly, lyophilized *Arabidopsis* seedlings (four days in AIC) were soaked with 2 M HCl (1 mg dry weight in 10 μL) and incubated at 100 °C for 20 min. Half volume of isoamylalcohol (1 mg dry weight in 5 μL) was then mixed to extract and concentrate aglycone flavonoids (top layer after centrifugation). The amount of flavonoids in each sample was detected by multiple reaction monitoring (MRM) and quantified by comparing with flavonoid standards on high-performance reverse phase liquid chromatography coupled with triple quadruple mass spectrometer^63^ using Waters MassLynx v4.1 SCN805. For creating standard curves of flavonoid compounds, the flavonoid standards were dissolved in 80% of methanol with 0.1% formic acid. The UHPLC-MS/MS run was 10 min with a flow of 0.3 ml/min at 40 °C. The gradient was as follows with solvent A (0.1% formic acid in water) and solvent B (0.1% formic acid in acetonitrile): 0–0.5 min, 90% A and 10% B; 0.5–6 min, 50% A and 50% B; 6–8 min, 0% A and 100% B; and 8.01–10 min 90% A and 10% B.

### Untargeted metabolite profiling and LC-MS/MS data analysis

For extraction of total metabolites, lyophilized *Arabidopsis* seedlings (four days in AIC or water) were incubated with methanol/water (1/1, v/v) containing 0.1% formic acid and 1 μM telmisartan (internal standard) at the ratio 1 mg dry weight in 10 μL extraction buffer overnight at room temperature. After centrifugation at 10,000 xg for 5 min, the supernatant was then diluted ten times with 80% of methanol with 0.1% formic acid for LC-MS/MS analysis.

All the genotypes were analyzed in triplicate by LC-MS/MS using Acquity UHPLC system with HSS T3 C18 column (100 × 2.1 mm, 1.8 μM, Waters, Milford, MA) linked to the mass spectrometer (Xevo G2-XS QTOF, Waters). At a flow rate of 0.3 mL/min and the column temperature of 40 °C, a 10 min gradient run was applied with solvent A (0.1% formic acid in water) and solvent B (acetonitrile): 0–0.5 min, 100% A; 0.5–6 min, 50% A and 50% B; 6.01–6.5 min, 1% A and 99% B; 6.51–8.5 min, 1% A and 99% B; and 8.51–10 min 99% A and 1% B. Mass spectrum acquistion was performed in negative mode with m/z scan range at 50–2000.

All MS raw data files (.raw) were converted to mzXML format using ProteoWizard version 3^64^ and then uploaded to XCMS online^65,66^ (https://xcmsonline.scripps.edu/) for data processing. For all the samples, peak alignment (feature detection), candidate metabolite identification, MS/MS matching, and statistical analysis were performed. Feature detection and pairwise analysis were performed with the following paramerters: centWave method for feature detection (Δm/z = 15 ppm, minimum peak width = 5 s, and maximun peak width = 50 s); obiwarp method for retention time correction (profStep = 1); and settings for alignment (bw = 5, minfrac = 0.5, mzwid = 0.015) and statistics (Welch *t*-test). The total numbers of aligned features and pairwise cloud plots were exported from XCMS online and reported in Supplementary Data 1 and Supplementary Fig. 8, respectively.

### Quantitative RT-PCR

Total RNA was extracted using QIAGEN RNeasy Plant Mini Kit. First-strand cDNA was synthesized from 1.5 μg of RNA using SuperScript II Reverse Transcriptase (ThermoFisher Scientific) and then diluted 1:5 with nuclease-free water. Quantitative PCR was performed in a 10 μL of mixture including 5 μL of SYBR Green PCR Master Mix (ThermoFisher Scientific), 1.2 μL of gene specific primer pair (0.6 μL of each 5 μM forward and reverse primers), and 2 μL of the cDNA samples. Quantitative PCR was conducted with the ABI QuantStudio 7 Flex PCR system (ThermoFisher Scientific) with the following cycling conditions: 50 °C for 2 min, 95 °C for 10 min, then followed by 40 cycles of 95 °C for 15 s, 58 °C for 10 s, and 72 °C for 30 s. PCR results were processed and visualized using QuantStudio Real-Time PCR software v1.3 (ThermoFisher Scientific). The experiments were performed in biological replicates (3 or more) and the housekeeping gene *Actin2* was used as the internal control for normalization of expression levels. The primer set used in this study is listed in Supplementary Table 2.

### Whole-genome re-sequencing

Total genomic DNA from pooled leaves of 50 *Arabidopsis* BC_1_F_3_ pigmented plants and 50 non-pigmented plants were extracted using Wizard Genomic DNA Purification Kit (Promega, WI). The pooled gDNA samples were then sent to BGI genomic services (http://www.genomics.cn/en/index) for 150 bp paired-end Illumina sequencing. The raw data quality check was done using FastQC (v0.11.5). Reads were aligned to the *Arabidopsis* reference genome (TAIR10) using Bowtie2 (v2.3.4). In total, approximately 340 million paired end reads were obtained. The alignment files were sorted and converted to BAM files using samtools (v1.6). The Mpileup tool in SAMtools was used for SNP calling and the conversion into VCF matrix was carried out using bcftools (v1.2). Variants were parsed using vcftools (v0.1.15) to include only high-quality single SNPs, which were defined as those with a minimum read depth of 5, a maximum read depth of 200 and a minimum mapping quality greater than 20 (–minDP 5;–MaxDP 200;–MinMQ 20;–remove-indels). SNP frequency was then calculated and used to filter each of the suppressor lines. Filter criteria was based on common SNPs in each suppressor line which had an allele frequency greater than 0.8 in the samples from pigmented plants and less than 0.4 in the corresponding samples from non-pigmented plants.

### RNA-seq analysis

Total RNA was extracted from *Arabidopsis* seedlings in AIC for four days using QIAGEN RNeasy Plant Mini Kit (Qiagen, Germany) according to the manufacturer’s instructions. Around 1 μg of RNA was used for construction of the RNA-seq libraries and sequenced with 100 bp paired-ends on an Illumina sequencing platform by BGI.

RNA-seq reads were evaluated for quality using FastQC (v.0.11.5). The reads were then aligned to the *Arabidopsis thaliana* reference genome (TAIR 10) using Hisat2 (v2.1). Raw read counts for gene features were then quantified from these alignments using FeatureCounts (v1.5). Transcript levels were calculated in transcripts per million (TPM). The R package DESeq2 (v1.24) was employed to test differential expression (DE) by paired contrast between *tt19-8* and Col-0 lines, and then between each suppressor lines against *tt19-8* and Col-0, and the resulting *P*-values were adjusted using the Benjamini and Hochberg’s method. Genes with an adjusted *P*-value <0.05 found by DESeq2 were assigned as differentially expressed, irrespectively of the fold change value, unless otherwise specified. The set of DE genes shared between different suppressor lines were assessed using a local R script and visualized using UpSetR package^67^.

Soft clustering was applied using Mfuzz (v2.44)^68^ on normalized centered gene expression values with an optimal fuzzifier value of 3.6 estimated from the total set of expressed genes (a total expression higher than 100 TPM using all the samples) to obtain a better view of the common behavior between the suppressor lines. The number of clusters was approximated by a built-in function and further adjusted until a suitable number of clusters appeared, i.e., without any empty group and with inter-cluster correlation value lower than 0.85.

### Small RNA-seq analysis

Total RNA was used to generate small RNA-seq library and sequenced with 50 bp single ends using the BGISEQ-500 platform. Adapter sequences were trimmed-off from the remaining high-quality sequences using Adapter Trimming for Small RNA-Sequencing tool (v0.3.2, http://www.bcgsc.ca/platform/bioinfo/software/adapter-trimming-for-small-RNA-sequencing) and sequences smaller than 15 nt were discarded. Unique small RNA tags were mapped to *Arabidopsis thaliana* reference genome (TAIR 10) using Bowtie (v1.1.2). In order to obtain miRNA abundance, we used the miRBase 22.1 database (http://www.mirbase.org/) annotations for *Arabidopsis thaliana*. The siRNA annotation was performed using compiled annotations from Araport11^41^.

In the global quantification analysis of small RNAs derived from miRNAs, *TAS* loci, transposable elements, and protein-coding genes, only small RNAs unique to single gene categories were included. For the quantification of small RNAs, we used a local Perl script for exact quantification, using loci length and read size restrictions depending of the small RNA category analyzed. For read normalization and differential expression analysis of small RNAs, we used edgeR (v3.26.5) to apply trimmed means of M-values as a normalization method (TMM) with FDR threshold value less than 0.05.

### Statistical analyses

Statistical analyses were conducted by GraphPad Prism 6.0c software (San Diego, California).

### Reporting summary

Further information on research design is available in the Nature Research Reporting Summary linked to this article.

## Supplementary information

Supplementary Information

Peer Review

Reporting Summary

Description of Additional Supplementary Files

Supplementary Data 1

Supplementary Data 2

Supplementary Data 3

Supplementary Data 4

Supplementary Data 5

Supplementary Data 6

## Data Availability

Data supporting the findings of this work are available within the paper and the respective Supplementary Information files. A reporting summary for this article is available as a Supplementary Information file. The datasets generated and analyzed during the current study are available from the corresponding author upon request. The raw reads of RNA-seq and small RNA-seq were deposited in the NCBI Gene Expression Omnibus (GEO) and whole-genome re-sequencing data were uploaded to Sequence Read Archive (SRA) under accession numbers GSE136680 [https://www.ncbi.nlm.nih.gov/geo/query/acc.cgi?acc=GSE136680], GSE136901 [https://www.ncbi.nlm.nih.gov/geo/query/acc.cgi?acc=GSE136901], and PRJNA562949 [https://www.ncbi.nlm.nih.gov/Traces/study/?acc=PRJNA562949], respectively. Source data underlying Figs. 2, 3, and 5, and Supplementary Figs 6, 7, 9, 10b, c, e, 12a, b, and 13 are provided as a Source Data file.

## Source Data

Source Data
