# Author Correction: Synergy between the anthocyanin and RDR6/SGS3/DCL4 siRNA pathways expose hidden features of *Arabidopsis* carbon metabolism

**DOI:** 10.1038/s41467-020-19223-9

**Published:** 2020-10-14

**Authors:** Nan Jiang, Aimer Gutierrez-Diaz, Eric Mukundi, Yun Sun Lee, Blake C. Meyers, Marisa S. Otegui, Erich Grotewold

**Affiliations:** 1 Department of Biochemistry and Molecular Biology, Michigan State University, East Lansing, MI 48824 USA; 2 Donald Danforth Plant Science Center, St. Louis, MO 63132 USA; 3 Division of Plant Sciences, University of Missouri, Columbia, MO 65201 USA; 4 Department of Botany, Laboratory of Molecular and Cell Biology, University of Wisconsin-Madison, Wisconsin-Madison, WI 53706 USA

**Keywords:** Metabolic engineering, Epigenetics, Plant genetics, Secondary metabolism

Correction to: *Nature Communications* 10.1038/s41467-020-16289-3, published online 15 May 2020.

The original version of the Supplementary Information associated with this Article contained errors in Supplementary Figs. 8 and 10. In Supplementary Fig. 8, the axes and the grid lines were missing from all six panels. In panel d of Supplementary Fig. 10, the figure element linking the sequences to the gene structure diagram was missing. The HTML has been updated to include a corrected version of the Supplementary Information.

