# Supplementary material for: Synergy between the anthocyanin and RDR6/SGS3/DCL4 siRNA pathways expose hidden features of Arabidopsis carbon metabolism
Source: Nat Commun. 2020 May 15;11:2456. doi: 10.1038/s41467-020-16289-3 (PMC7229025; doi:10.1038/s41467-020-16289-3)
Supplement: Supplementary file 1 — Supplementary Information [file 41467_2020_16289_MOESM1_ESM.pdf]

Synergy between the anthocyanin and RDR6/SGS3/DCL4 siRNA pathways expose hidden features of *Arabidopsis* carbon metabolism

Jiang *et al.*

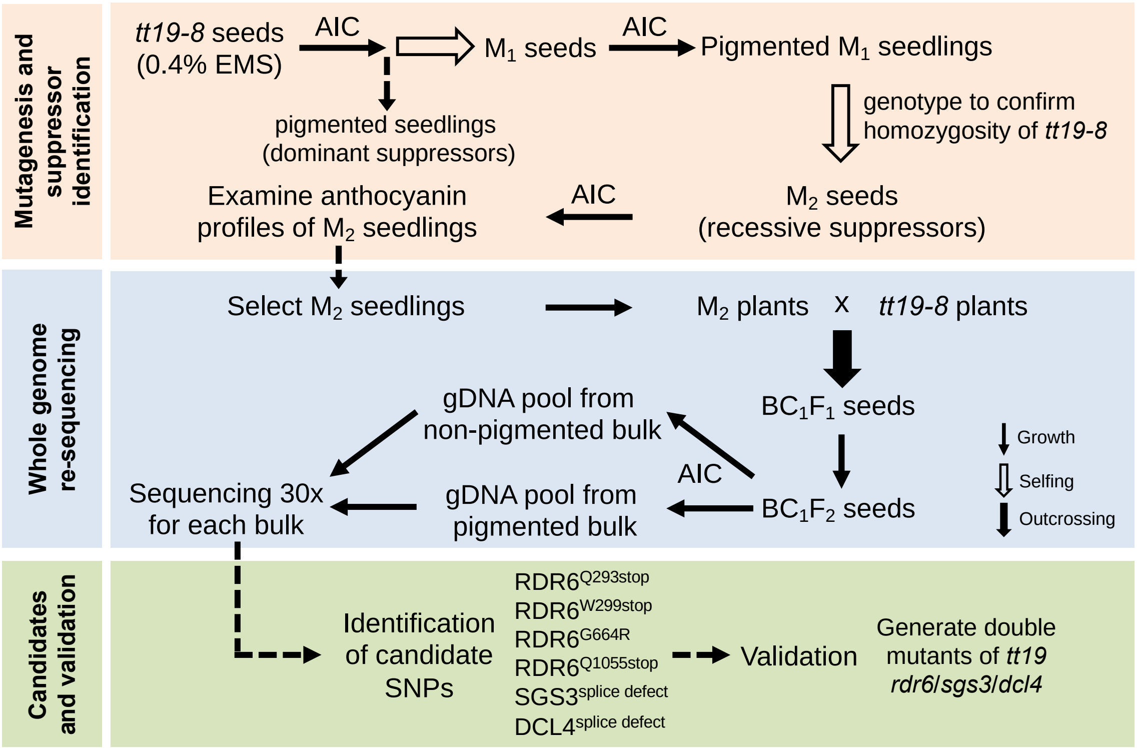

Supplementary Figure 1. Flow chart to identify EMS mutagenized mutations as *tt19* mutant suppressors on anthocyanin restoration using whole genome re-sequencing.

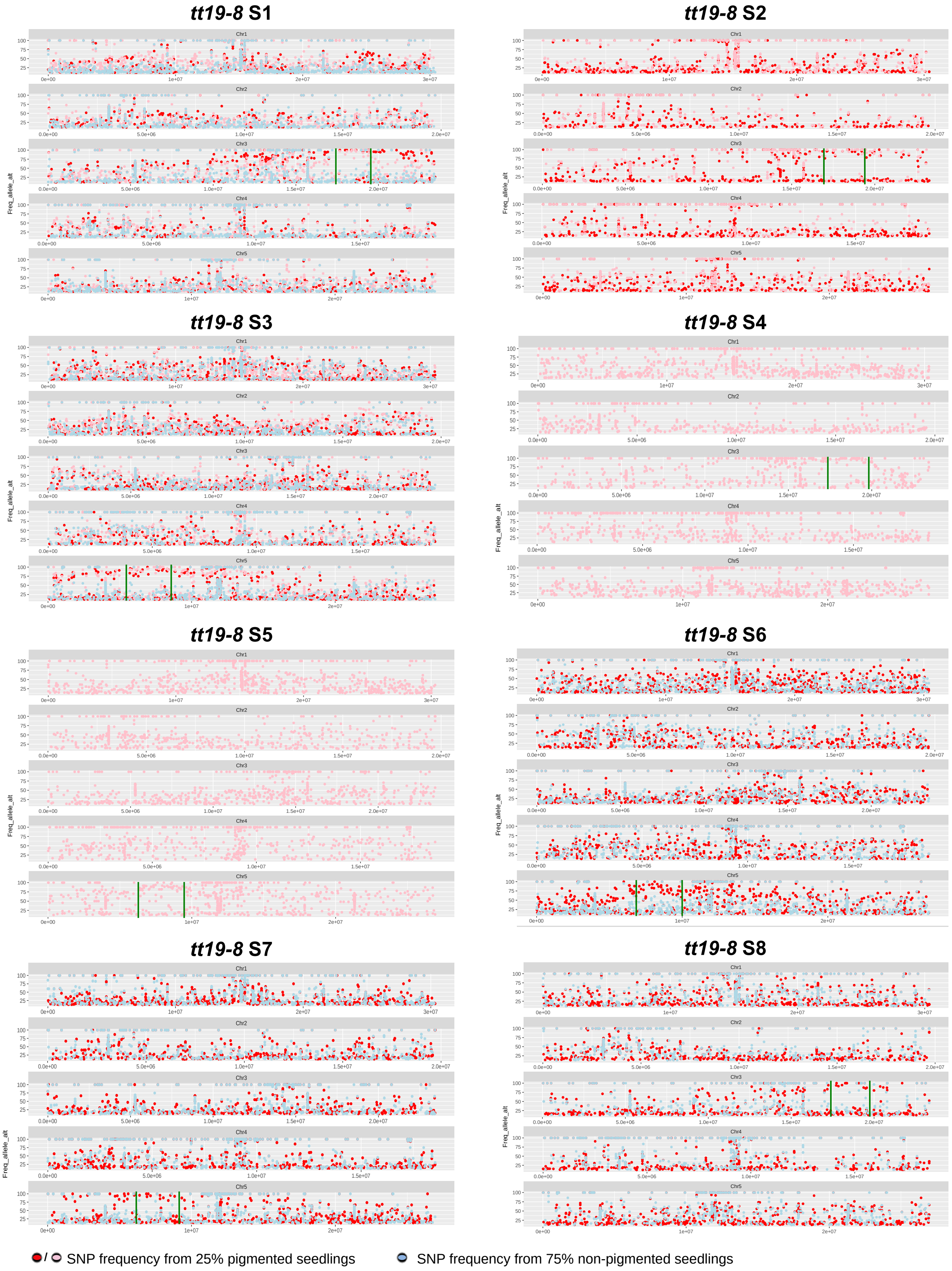
tt19-8 S1
tt19-8 S2
tt19-8 S3
tt19-8 S4
tt19-8 S5
tt19-8 S6
tt19-8 S7
tt19-8 S8
Chr1
Chr2
Chr3
Chr4
Chr5
Freq_allele_alt
SNP frequency from 25% pigmented seedlings
SNP frequency from 75% non-pigmented seedlings

Supplementary Figure 2. Whole genome re-sequencing of bulked segregants of all eight *tt19* suppressor lines. The *y*-axis indicates the allelic frequencies of a pool of 50 pigmented seedlings (represented by red and pink circles where two samples were used or pink circle where only one sample was used) or 50 non-pigmented seedlings (represented by blue circles) at genome locations (*x*-axis) corresponding to each of the five *Arabidopsis* chromosomes (one in each line). The *tt19-8* S2, S4, and S5 samples comprise of only sequenced pools of pigmented seedlings. Green bars highlighted the candidate regions contain causative mutations. Candidate SNPs in *tt19* suppressors S1, S2, S4, and S8 are located at the bottom arm of chromosome 3. For S3, S5, S6, and S7, two candidate SNPs were in the upper arm of chromosome 5.

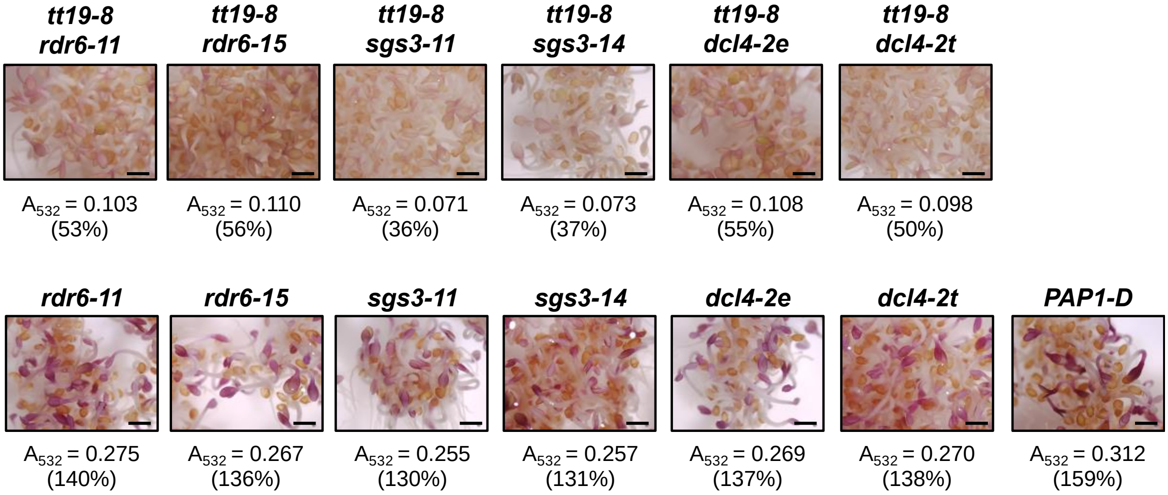

Supplementary Figure 3. Anthocyanin accumulation phenotype of 4-day-old seedlings grown in AIC for various single and double mutants. The % values correspond to the amounts of anthocyanins in methanol extracts with respect to Col-0 (100%) estimated by A_{532} and normalized to dry weight. Similar anthocyanin induced results were obtained from at least three independently repeated experiments. Bar, 400 µm.

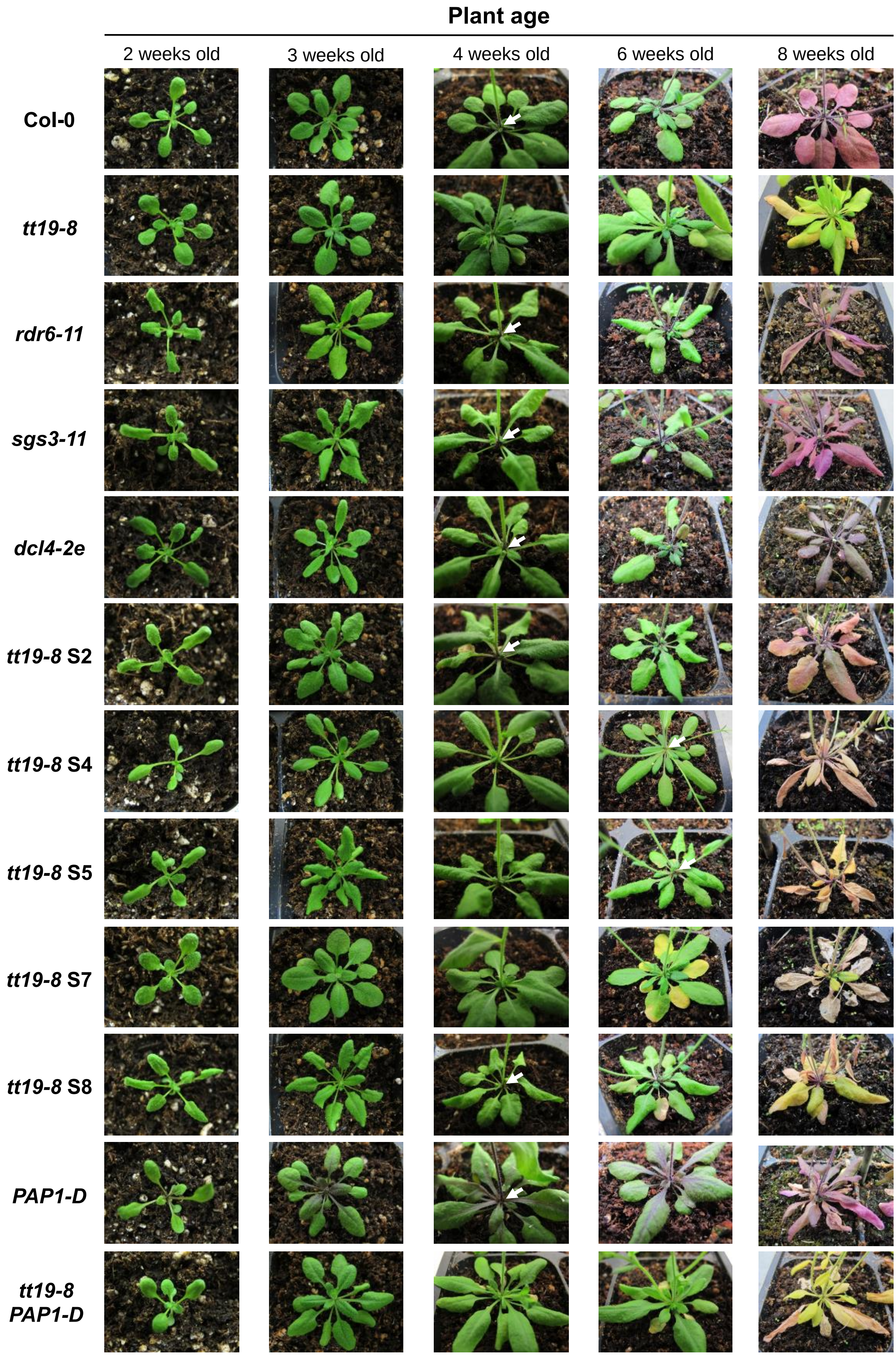
Plant age
2 weeks old
3 weeks old
4 weeks old
6 weeks old
8 weeks old
Col-0
tt19-8
rdr6-11
sgs3-11
dcl4-2e
tt19-8 S2
tt19-8 S4
tt19-8 S5
tt19-8 S7
tt19-8 S8
PAP1-D
tt19-8 PAP1-D

Supplementary Figure 4. Anthocyanin accumulation in plants of various ages harboring the various *tt19-8* suppressor alleles as well as the reference and *PAP1-D* alleles. Visible anthocyanin accumulation was observed at the base of the inflorescence stem in the *rdr6-11*, *sgs3-11* and *decl4-e2* single mutant plants, and in the *tt19* suppressor lines S2 and S8 at around four weeks after germination, but never in *tt19-8* plants. At six weeks, anthocyanin pigmentation was detected in lines S4 and S5. No obvious anthocyanin pigmentation was observed in adult plants of *tt19-8* S7. White arrows in four- or six-week old plants indicate the sites where anthocyanin accumulated in Col-0, *rdr6-11*, *sgs3-11*, *dcl4-2e*, *tt19-8* S2, *tt19-8* S4, *tt19-8* S5, *tt19-8* S8, and *PAP1-D*.

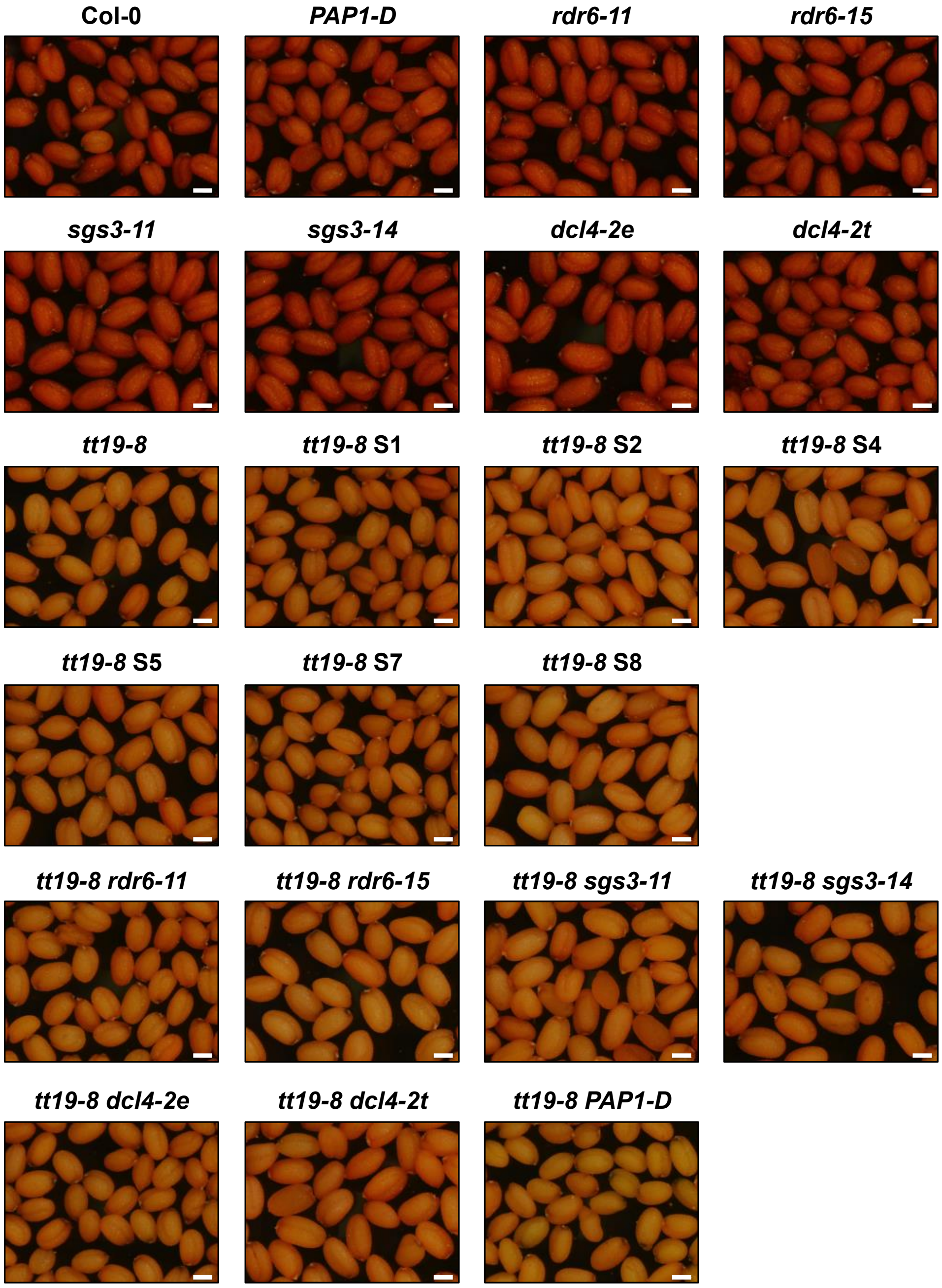

Supplementary Figure 5. Phenotypes of seed coat colors in Col-0 and various mutant alleles. Bar, 200 μm.

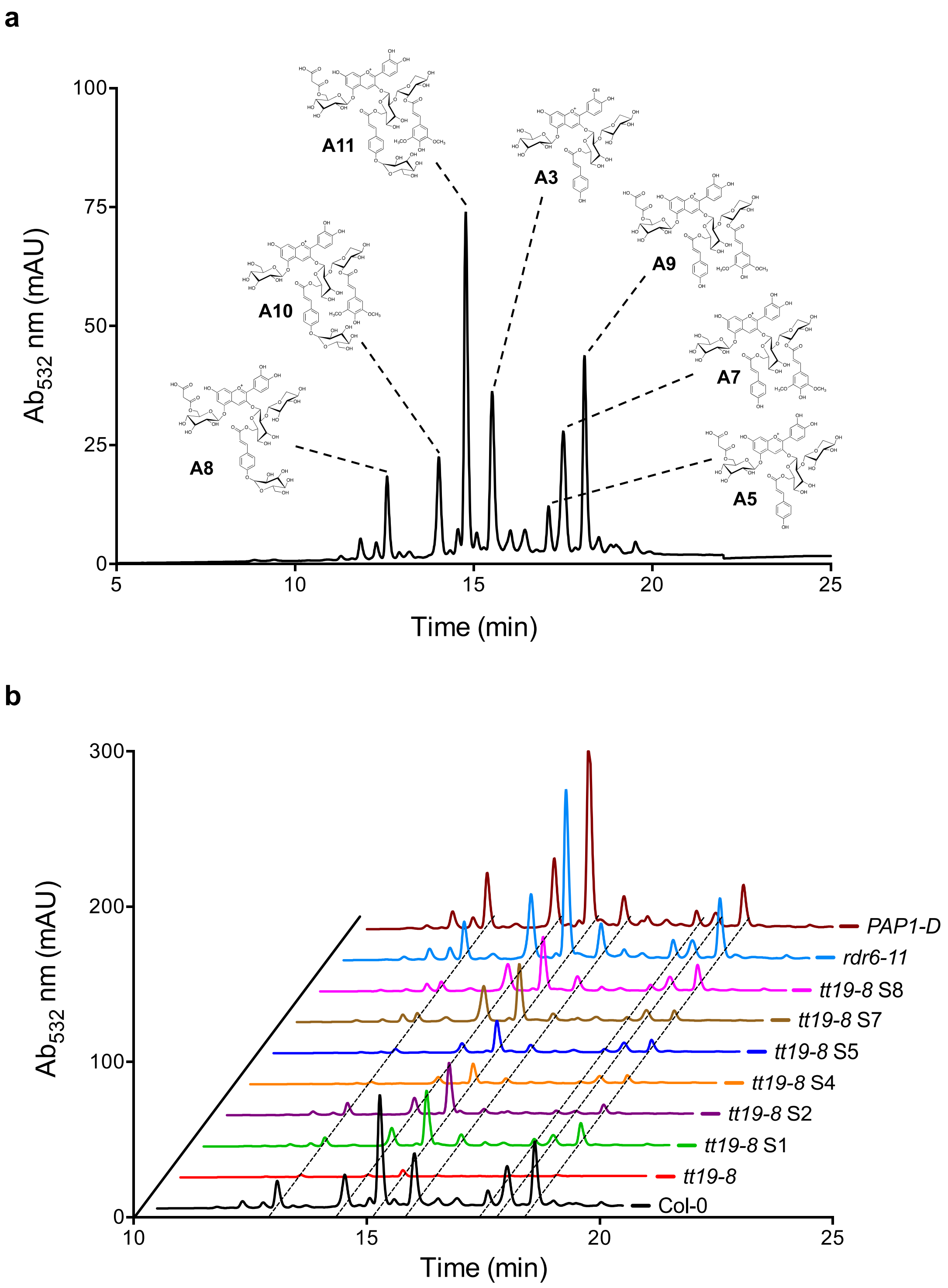

Supplementary Figure 6. Anthocyanin profiles of Col-0 and *tt19* suppressors. Four day-old seedlings were grown in AIC, anthocyanins were extracted and separated by HPLC. **(a)** Anthocyanin HPLC profile of Col-0 seedlings, in which major peaks were confirmed by LC-MS/MS. **(b)** Distribution of anthocyanin peaks compared among Col-0 and various mutants. Dashed lines from left to right correspond to the peaks (A8, A10, A11, A3, A5, A7, and A9) indicate in (a). Source data are provided as a Source Data file.

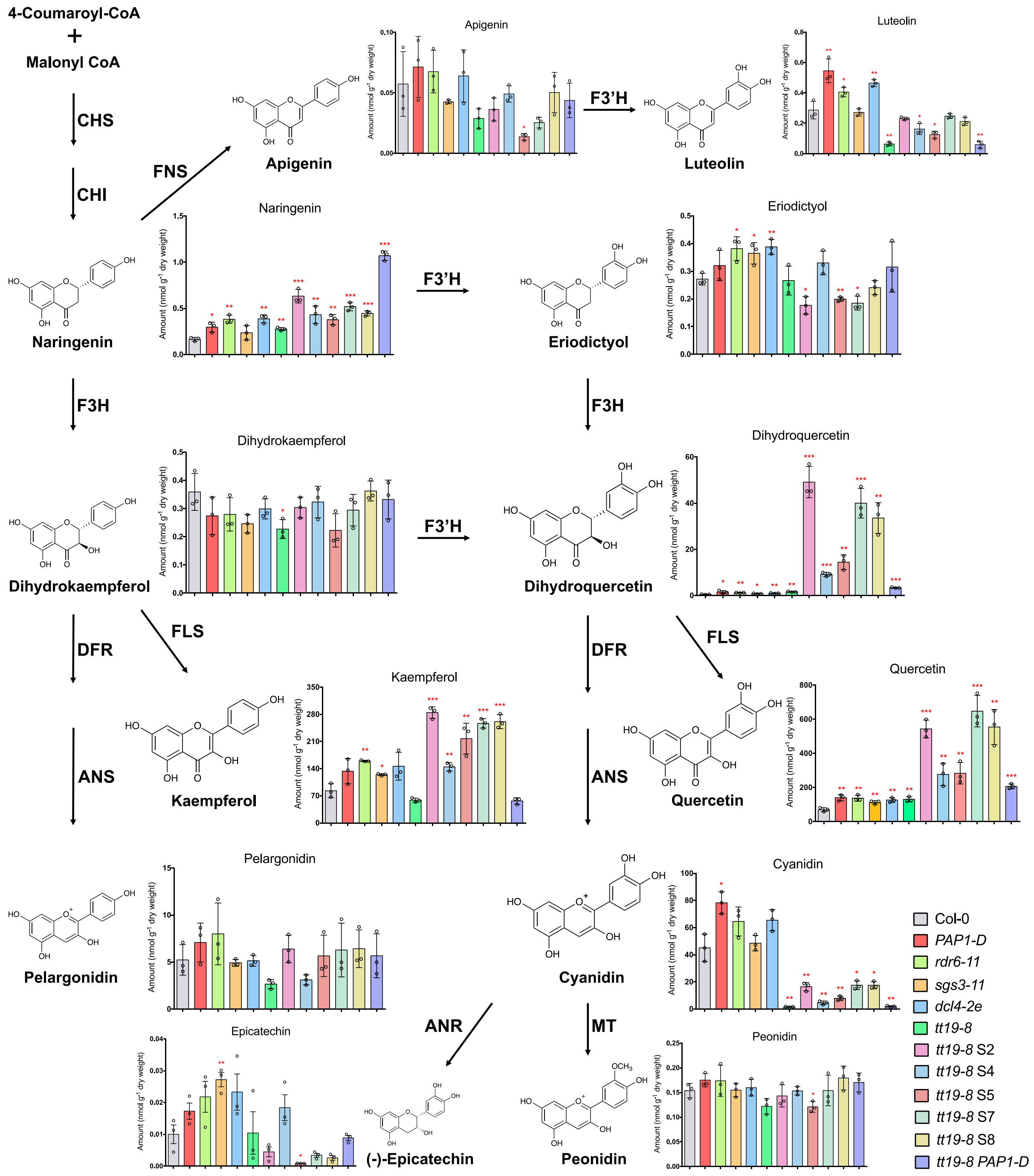

Supplementary Figure 7. Accumulation of *Arabidopsis* flavonoids in Col-0 and *tt19* suppressors. A summary of this figure (without the pathway) is provided as Fig. 3. The accumulation of all pathway intermediates and final products were quantified by LC-MS/MS against authentic standards of known concentration in 4-day-old seedlings grown in AIC for each of the genotypes indicated. The experiments were performed in biological triplicate. The error bars represent the standard deviation of the average. The stars indicate a statistically (paired two-tailed *t*-test) significant (* $P < 0.05$, ** $P < 0.01$, and *** $P < 0.001$) difference to Col-0. Source data are provided as a Source Data file.

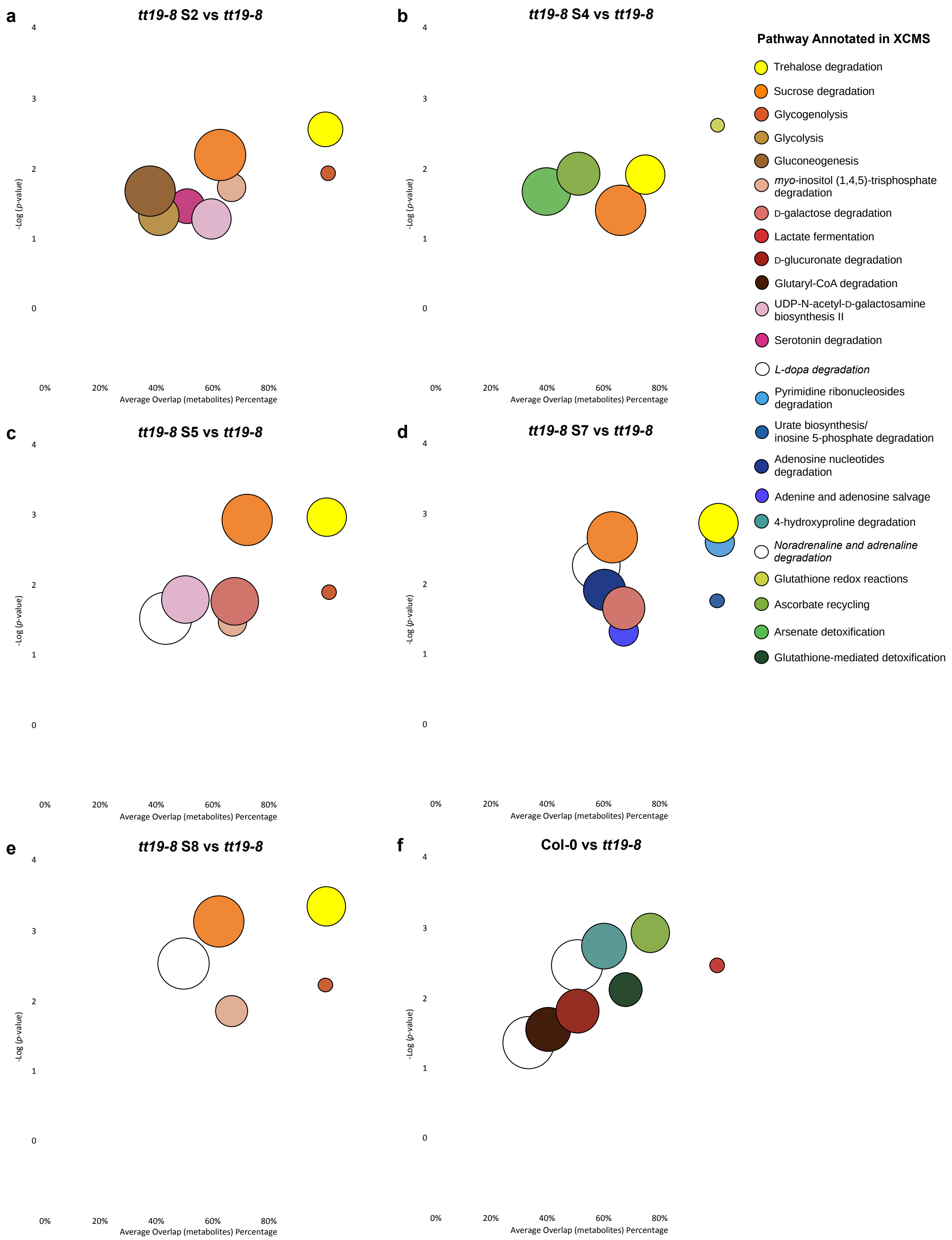
a
tt19-8 S2 vs tt19-8
b
tt19-8 S4 vs tt19-8
c
tt19-8 S5 vs tt19-8
d
tt19-8 S7 vs tt19-8
e
tt19-8 S8 vs tt19-8
f
Col-0 vs tt19-8
-Log (p-value)
0
1
2
3
4
0%
20%
40%
60%
80%
Average Overlap (metabolites) Percentage
Pathway Annotated in XCMS
Trehalose degradation
Sucrose degradation
Glycogenolysis
Glycolysis
Gluconeogenesis
myo-inositol (1,4,5)-trisphosphate degradation
D-galactose degradation
Lactate fermentation
D-glucuronate degradation
Glutaryl-CoA degradation
UDP-N-acetyl-D-galactosamine biosynthesis II
Serotonin degradation
L-dopa degradation
Pyrimidine ribonucleosides degradation
Urate biosynthesis/ inosine 5-phosphate degradation
Adenosine nucleotides degradation
Adenine and adenosine salvage
4-hydroxyproline degradation
Noradrenaline and adrenaline degradation
Glutathione redox reactions
Ascorbate recycling
Arsenate detoxification
Glutathione-mediated detoxification

Supplementary Figure 8. Metabolic pathway cloud plot representation of metabolic alterations in *tt19* suppressor lines. The results correspond to pairwise comparisons between the *tt19* suppressor lines *tt19-8* S2 (a), S4 (b), S5 (c), S7 (d), S8 (e), and Col-0 (f) with *tt19-8*. The plots show the results of dysregulated pathways after statistical significance filtering (*P*-value < 0.05) for each aligned feature. Each colored circle displays a predictive pathway with the radius of the circle representing the size of the pathway (*x*-axis for the percentage of overlapped metabolites and *y*-axis for statistical significance). Significantly dysregulated candidate pathways are shown on the upper right portion of the plot. Note: Some of the pathways with italics do not necessarily exist in plants.

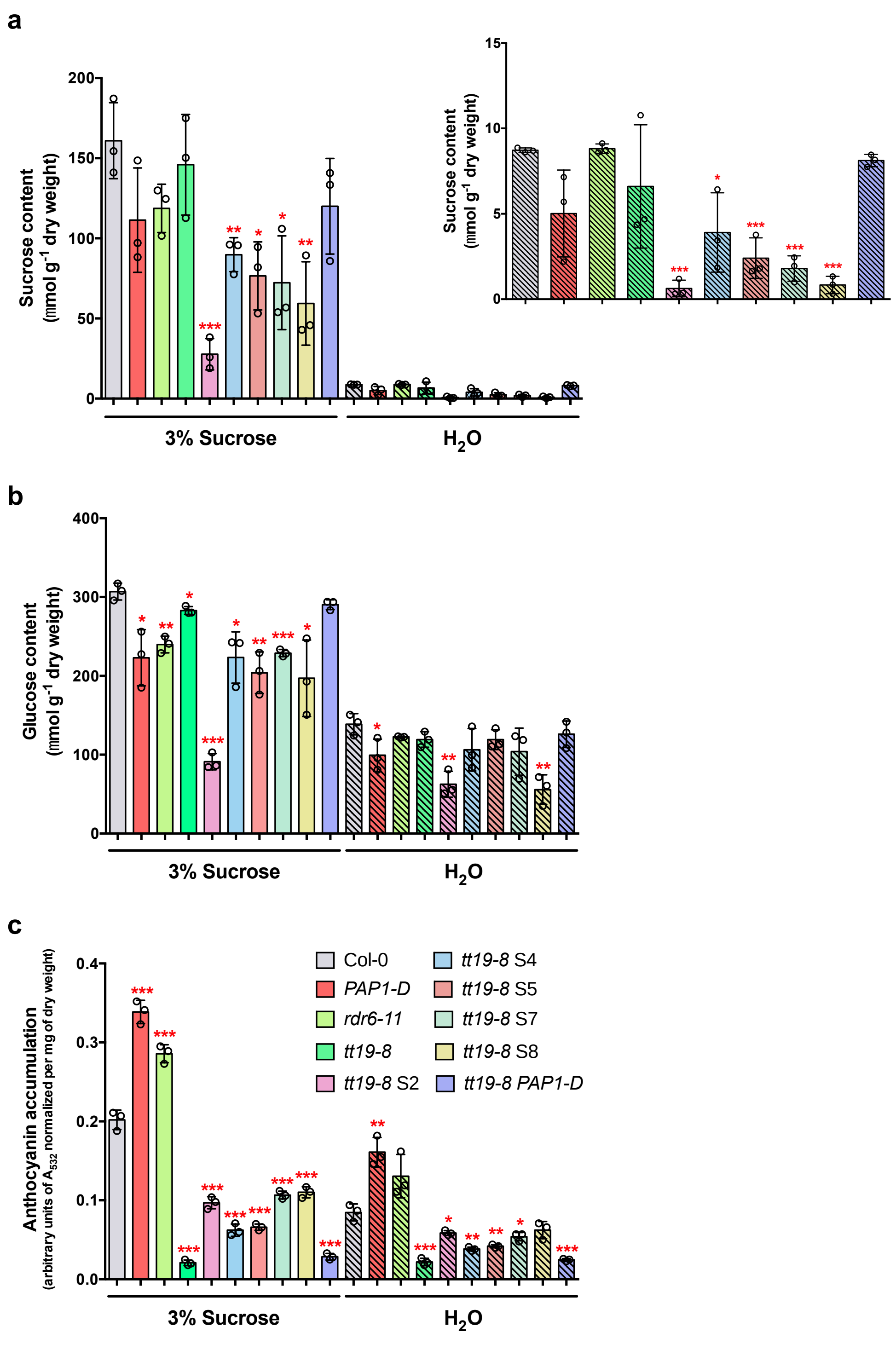

Supplementary Figure 9. Sugar accumulation in Col-0 and *tt19* suppressors. Measurement correspond to levels of sucrose (a), glucose (b), and anthocyanins (c) in 4-day-old *Arabidopsis* seedlings. Seedlings were grown in AIC (3% sucrose, clear columns) or water (H_2O, shaded columns) for each of the genotypes with metabolite quantification performed by LC-MS/MS against authentic standards of known concentration and absorbance at 532 nm (normalized per mg of dry weight). The experiments were performed in biological triplicate and the error bars represent the standard deviations of the averages. The stars indicate a statistically (paired two-tailed *t*-test) significant (* $P < 0.05$, ** $P < 0.01$, and *** $P < 0.001$) difference to Col-0 within the same treatment group. Source data are provided as a Source Data file.

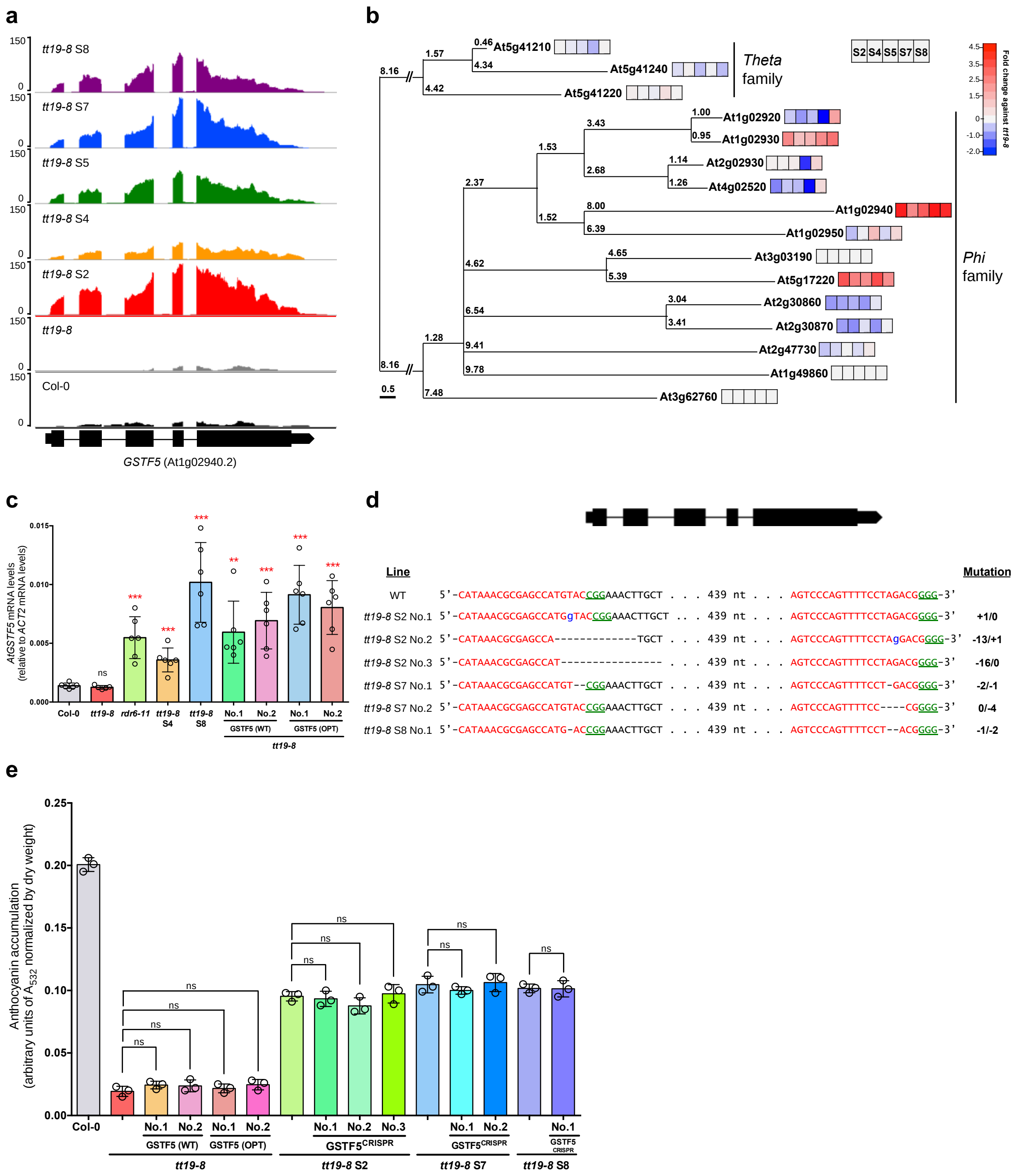
a
tt19-8 S8
tt19-8 S7
tt19-8 S5
tt19-8 S4
tt19-8 S2
tt19-8
Col-0
GSTF5 (At1g02940.2)
b
Theta family
Phi family
S2 S4 S5 S7 S8
Fold change against tt19-8
At5g41210
At5g41240
At5g41220
At1g02920
At1g02930
At2g02930
At4g02520
At1g02940
At1g02950
At3g03190
At5g17220
At2g30860
At2g30870
At2g47730
At1g49860
At3g62760
c
AtGSTF5 mRNA levels (relative to ACT2 mRNA levels)
Col-0
tt19-8
rdr6-11
tt19-8 S4
tt19-8 S8
No.1
No.2
GSTF5 (WT)
GSTF5 (OPT)
d
Line
Mutation
WT 5'-CATAAACGCGAGCCATGTACCGGAAACTTGCT . . . 439 nt . . . AGTCCCAGTTTTCCTAGACGGGG-3'
tt19-8 S2 No.1 5'-CATAAACGCGAGCCATGgTACCGGAAACTTGCT . . 439 nt . . . AGTCCCAGTTTTCCTAGACGGGG-3' +1/0
tt19-8 S2 No.2 5'-CATAAACGCGAGCCA-------------TGCT . . . 439 nt . . . AGTCCCAGTTTTCCTAgGACGGGG-3' -13/+1
tt19-8 S2 No.3 5'-CATAAACGCGAGCCAT---------------- . . . 439 nt . . . AGTCCCAGTTTTCCTAGACGGGG-3' -16/0
tt19-8 S7 No.1 5'-CATAAACGCGAGCCATGT--CGGAAACTTGCT . . . 439 nt . . . AGTCCCAGTTTTCCT-GACGGGG-3' -2/-1
tt19-8 S7 No.2 5'-CATAAACGCGAGCCATGTACCGGAAACTTGCT . . . 439 nt . . . AGTCCCAGTTTTCC----CGGGG-3' 0/-4
tt19-8 S8 No.1 5'-CATAAACGCGAGCCATG-ACCGGAAACTTGCT . . . 439 nt . . . AGTCCCAGTTTTCCT--ACGGGG-3' -1/-2
e
Anthocyanin accumulation (arbitrary units of A532 normalized by dry weight)
Col-0
No.1 No.2 No.1 No.2
GSTF5 (WT) GSTF5 (OPT)
tt19-8
No.1 No.2 No.3
GSTF5CRISPR
tt19-8 S2
No.1 No.2
GSTF5CRISPR
tt19-8 S7
No.1
GSTF5 CRISPR
tt19-8 S8
ns

Supplementary Figure 10. AtGSTF5 is not involved in anthocyanin accumulation in *tt19* mutant suppressors. (a) The mapped reads of RNA-seq analysis across *AtGSTF5* region were compared among Col-0, *tt19-8*, and *tt19* suppressors. **(b)** Phylogenetic tree between *Arabidopsis Theta* and *Phi* family, constructed using the neighbor-joining method. Differential expression levels of genes in the GSTT and GSTF family were represented as fold changes between *tt19* suppressors and *tt19-8*. **(c)** RT-qPCR analysis to measure *AtGSTF5* mRNA levels (normalized to *ACT2* mRNAs) among Col-0, *tt19-8*, *tt19* suppressors, and *AtGSTF5* overexpressed transgenic lines. n = 6, 5, 6, 6, 6, 6, 6, 6, 6 biological replicates. **(d)** *AtGSTF5* structure with two guide DNA target sites for CRISPR. Red color highlights two target sites, and green underlines indicate PAM sequences. Site-specific mutations in *tt19* suppressor lines were confirmed by sequencing. The nucleotide changes (dashes indicate deletions and blue lowercase letters mean insertions) in two target sites were calculated on the right side of each sequence. **(e)** Anthocyanin levels were measured as A_{532} of 4-day-old seedlings in AIC of Col-0, *tt19-8*, *tt19-8* overexpressed GSTF5, *tt19* suppressors, and *tt19* suppressors with *AtGSTF5* CRISPR lines. The experiments were performed in biological triplicate. The error bars represent the standard deviation of the average. The stars indicate a statistically (paired two-tailed *t*-test) significant (** $P < 0.01$ and *** $P < 0.001$) difference to wild-type Col-0. ns, not significant. Source data underlying Supplementary Figure 10b, 10c, and 10e are provided as a Source Data file.

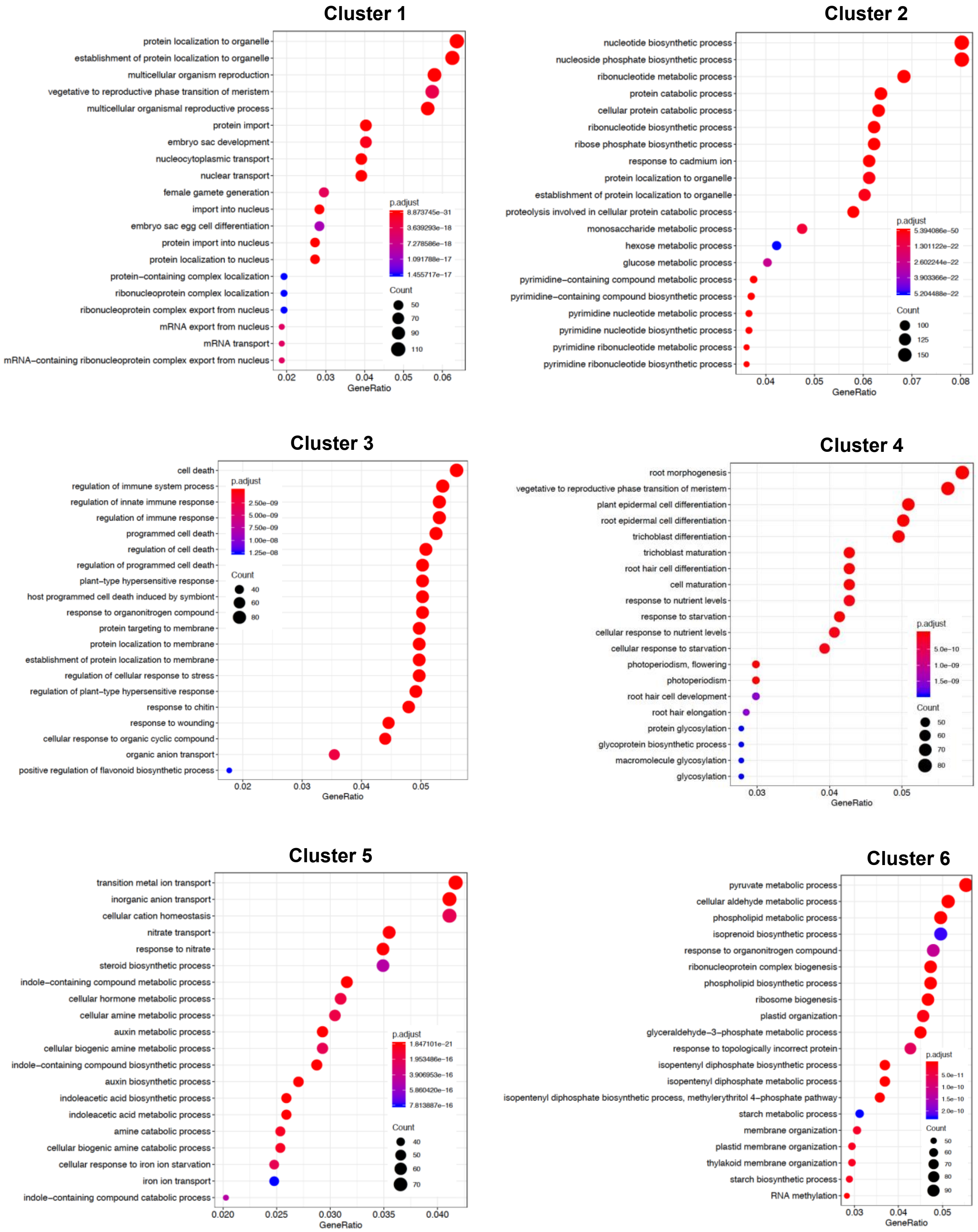

Cluster 1
protein localization to organelle
establishment of protein localization to organelle
multicellular organism reproduction
vegetative to reproductive phase transition of meristem
multicellular organismal reproductive process
protein import
embryo sac development
nucleocytoplasmic transport
nuclear transport
female gamete generation
import into nucleus
embryo sac egg cell differentiation
protein import into nucleus
protein localization to nucleus
protein−containing complex localization
ribonucleoprotein complex localization
ribonucleoprotein complex export from nucleus
mRNA export from nucleus
mRNA transport
mRNA−containing ribonucleoprotein complex export from nucleus
p.adjust
8.873745e−31
3.639293e−18
7.278586e−18
1.091788e−17
1.455717e−17
Count
50
70
90
110
0.02
0.03
0.04
0.05
0.06
GeneRatio
Cluster 2
nucleotide biosynthetic process
nucleoside phosphate biosynthetic process
ribonucleotide metabolic process
protein catabolic process
cellular protein catabolic process
ribonucleotide biosynthetic process
ribose phosphate biosynthetic process
response to cadmium ion
protein localization to organelle
establishment of protein localization to organelle
proteolysis involved in cellular protein catabolic process
monosaccharide metabolic process
hexose metabolic process
glucose metabolic process
pyrimidine−containing compound metabolic process
pyrimidine−containing compound biosynthetic process
pyrimidine nucleotide metabolic process
pyrimidine nucleotide biosynthetic process
pyrimidine ribonucleotide metabolic process
pyrimidine ribonucleotide biosynthetic process
p.adjust
5.394086e−50
1.301122e−22
2.602244e−22
3.903366e−22
5.204488e−22
Count
100
125
150
0.04
0.05
0.06
0.07
0.08
GeneRatio
Cluster 3
cell death
regulation of immune system process
regulation of innate immune response
regulation of immune response
programmed cell death
regulation of cell death
regulation of programmed cell death
plant−type hypersensitive response
host programmed cell death induced by symbiont
response to organonitrogen compound
protein targeting to membrane
protein localization to membrane
establishment of protein localization to membrane
regulation of cellular response to stress
regulation of plant−type hypersensitive response
response to chitin
response to wounding
cellular response to organic cyclic compound
organic anion transport
positive regulation of flavonoid biosynthetic process
p.adjust
2.50e−09
5.00e−09
7.50e−09
1.00e−08
1.25e−08
Count
40
60
80
0.02
0.03
0.04
0.05
GeneRatio
Cluster 4
root morphogenesis
vegetative to reproductive phase transition of meristem
plant epidermal cell differentiation
root epidermal cell differentiation
trichoblast differentiation
trichoblast maturation
root hair cell differentiation
cell maturation
response to nutrient levels
response to starvation
cellular response to nutrient levels
cellular response to starvation
photoperiodism, flowering
photoperiodism
root hair cell development
root hair elongation
protein glycosylation
glycoprotein biosynthetic process
macromolecule glycosylation
glycosylation
p.adjust
5.0e−10
1.0e−09
1.5e−09
Count
50
60
70
80
0.03
0.04
0.05
GeneRatio
Cluster 5
transition metal ion transport
inorganic anion transport
cellular cation homeostasis
nitrate transport
response to nitrate
steroid biosynthetic process
indole−containing compound metabolic process
cellular hormone metabolic process
cellular amine metabolic process
auxin metabolic process
cellular biogenic amine metabolic process
indole−containing compound biosynthetic process
auxin biosynthetic process
indoleacetic acid biosynthetic process
indoleacetic acid metabolic process
amine catabolic process
cellular biogenic amine catabolic process
cellular response to iron ion starvation
iron ion transport
indole−containing compound catabolic process
p.adjust
1.847101e−21
1.953486e−16
3.906953e−16
5.860420e−16
7.813887e−16
Count
40
50
60
70
0.020
0.025
0.030
0.035
0.040
GeneRatio
Cluster 6
pyruvate metabolic process
cellular aldehyde metabolic process
phospholipid metabolic process
isoprenoid biosynthetic process
response to organonitrogen compound
ribonucleoprotein complex biogenesis
phospholipid biosynthetic process
ribosome biogenesis
plastid organization
glyceraldehyde−3−phosphate metabolic process
response to topologically incorrect protein
isopentenyl diphosphate biosynthetic process
isopentenyl diphosphate metabolic process
isopentenyl diphosphate biosynthetic process, methylerythritol 4−phosphate pathway
starch metabolic process
membrane organization
plastid membrane organization
thylakoid membrane organization
starch biosynthetic process
RNA methylation
p.adjust
5.0e−11
1.0e−10
1.5e−10
2.0e−10
Count
50
60
70
80
90
0.03
0.04
0.05
GeneRatio

Supplementary Figure 11. Dot plots of GO term enrichment analyses on the six clusters shown in Fig. 4a. The GO processes with the 20 largest gene ratios in each cluster are plotted in order of gene ratio. Dot sizes represent the number of the significantly differential-expressed genes under each process and dot colors represent the *p*-adjusted values. The hypergeometric test was performed one-side with all subontologies, meaning biological process, molecular function, and cellular component. For enrichment analysis, a Benjamini-Hochberg correction was applied with a cutoff of 0.05. In cluster 2, 15 genes (At4g26850, At3g04120, At3g27300, At3g55440, At1g23190, At4g37870, At3g47800, At5g13110, At2g01140, At5g42740, At3g08590, At1g79550, At1g42970, At4g38970, and At3g43190) were enriched under the GO term *monosaccharide/hexose/glucose metabolic process* with two genes (At3g04120 and At4g38970) were also upregulated in *rdr6-11* mutant.

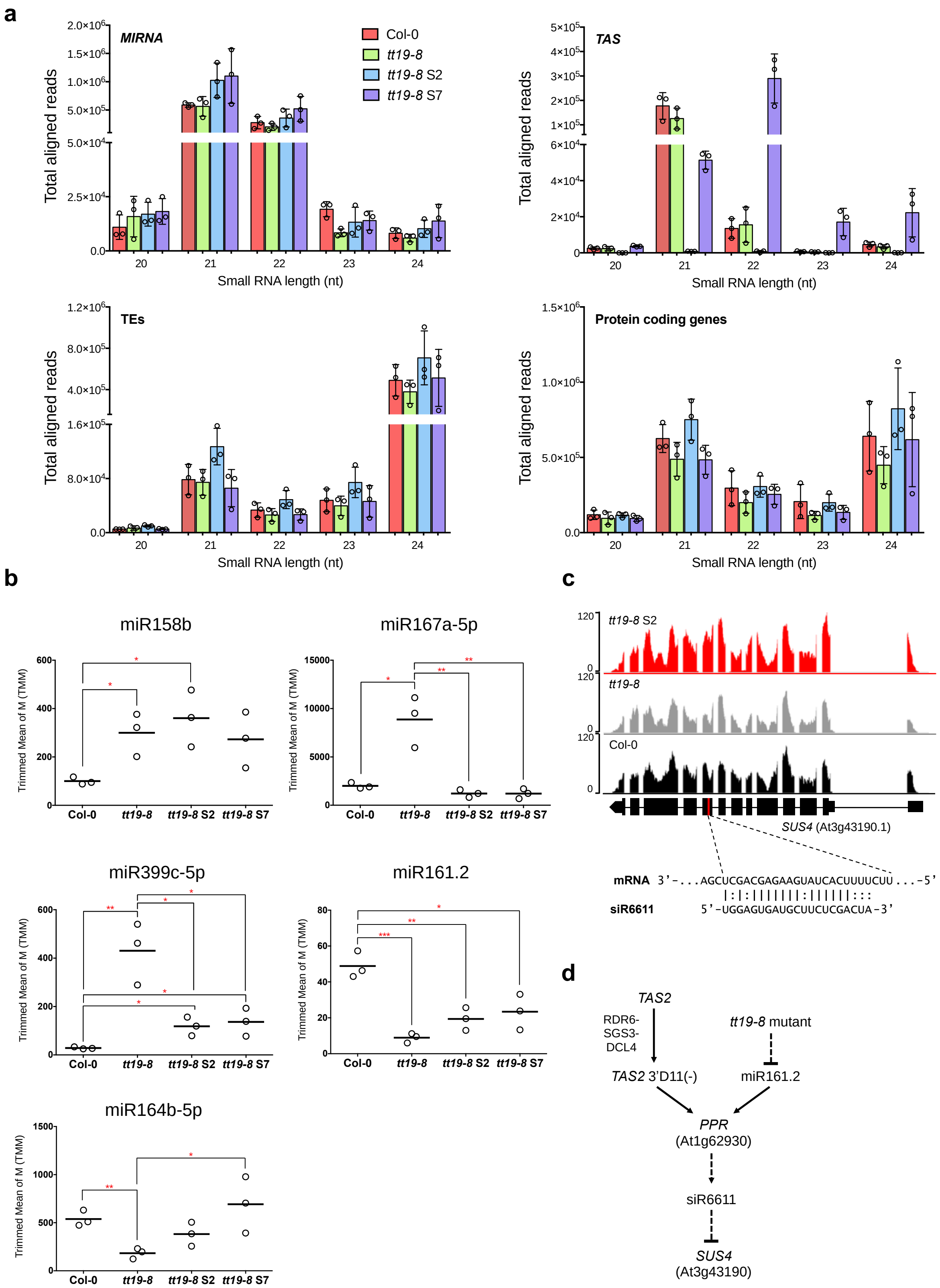
a
MIRNA
TAS
TEs
Protein coding genes
Col-0
tt19-8
tt19-8 S2
tt19-8 S7
Total aligned reads
Small RNA length (nt)
20
21
22
23
24
b
miR158b
miR167a-5p
miR399c-5p
miR161.2
miR164b-5p
Trimmed Mean of M (TMM)
Col-0
tt19-8
tt19-8 S2
tt19-8 S7
c
tt19-8 S2
tt19-8
Col-0
SUS4 (At3g43190.1)
mRNA 3'-...AGCUCGACGAGAAGUAUCACUUUUCUU...-5'
siR6611 5'-UGGAGUGAUGCUUCUCGACUA-3'
d
TAS2
RDR6-
SGS3-
DCL4
tt19-8 mutant
TAS2 3'D11(-)
miR161.2
PPR
(At1g62930)
siR6611
SUS4
(At3g43190)

Supplementary Figure 12. Small RNA-seq analysis for Col-0 and *tt19* suppressor lines. **(a)** Accumulation of total aligned reads of 20, 21, 22, 23, and 24 nt small RNAs derived from *MIRNA*, *TAS* loci, transposon elements (TEs), and protein coding genes seedlings grown in AIC. The experiments were performed in biological triplicate and the error bars represent the standard deviation of the average. **(b)** TT19 affects the expressions of five miRNAs (miR158b, miR161.2, miR167a-5p, miR399c-5p, and miR164b-5p). Open circles represent each biological sample and bars indicate the average of biological triplicates. The stars indicate a statistically (paired two-tailed *t*-test) significant (* $P < 0.05$, ** $P < 0.01$, and *** $P < 0.001$) difference. **(c)** Genome browser view of the mapped reads derived from RNA-seq across the *SUS4* region, compared between Col-0, *tt19-8*, and *tt19-8 S2*. The siRNA (siR6611) sequence aligned with the target sequence of *SUS4* is indicated. Base pairing is denoted by " | ", mismatches with " : ". **(d)** Proposed model for a synergistic effect between the *tt19-8* mutant and the RDR6-SGS3-DCL4 system on the cleavage of a *PPR* gene (At1g62930) by a miRNA (miR161.2) and a tasiRNA derived from *TAS2*, resulting in the regulation of *SUS4* mRNA accumulation by *PPR* gene-derived siR6611cleavage. Source data underlying Supplementary Figure 12a and 12b are provided as a Source Data file.

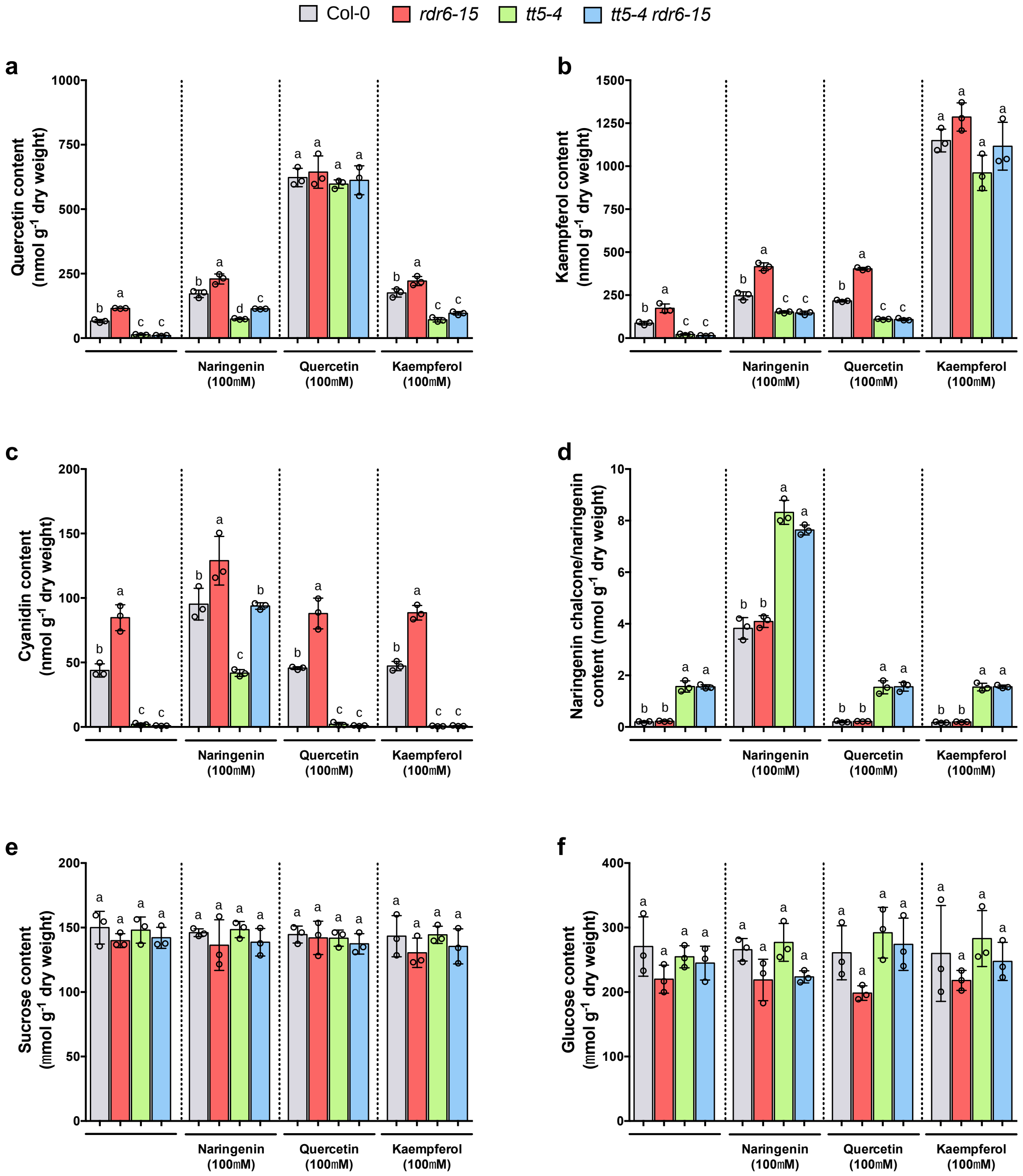

Supplementary Figure 13. Flavonoid levels in Col-0 and *tt19* suppressor lines. Measurement of quercetin (a), kaempferol (b), cyanidin (c), naringenin (d), sucrose (e), and glucose (f) accumulation in 4-day-old Col-0, *rdr6-15*, *tt5-4*, and *tt5-4 rdr6-15* seedlings. The seedlings were grown in AIC for three days and applied with 100 µM of naringenin/quercetin/kaempferol, respectively. The metabolite quantifications were performed by LC-MS/MS against authentic standards of known concentration. The experiments were performed in biological triplicate and the error bars represent the standard deviations of the averages. Different letters indicate significant differences between genotypes based on one-way ANOVA with Tukey's Honest Significant Difference test ($P < 0.05$). Source data are provided as a Source Data file.

Supplementary Table 1. Summary of the current *tt19* mutant alleles.

tt19 allele	Alternate name	Ecotype	Mutagen	Description of mutation	Phenotypes
tt19-1 [1,2]		Col-0	Ion beam irradiation	Inversion (~1000 kb) at intron 2	Pale brown seed coats;<br>Reduction of anthocyannins in seedlings
tt19-2 [3]		Col-0	Ion beam irradiation	Translocation (~16.7 kb) at position -53	
tt19-3 [3]			EMS	Missense, Ile to Phe at amini acid 70	
tt19-4 [3]	sk36391	Col-4	T-DNA	Missense, Trp to Leu at amini acid 205	Abolishes seed coat pigmentation;<br>Normal anthocyanin levels in vegetative tissues
tt19-5 [3]	sk4945	Col-4	T-DNA	Deletion (4 bp at 420-423), frameshift	
tt19-6 [3]	sk20780	Col-4	T-DNA	Insertion in first exon	
tt19-7 [4]		Col-0	EMS	Splice defect (donor loss between exon2/intron 2)	Light brown seed coat immediately after harvest, and seed coats become darker over time and indistinguishable from wild-type;<br>Reduction of anthocyannins in seedlings
tt19-8 [5, 6]	SALK_105779	Col-0	T-DNA	Insertion in second intron	Pale brown seed coats;<br>Reduction of anthocyannins in seedlings

Supplementary Table 2. List of primers.

Primer Name	Sequence	Purpose
tt19-8_geno_LP	TCAAAAGTGGTTGTTGGGAAG	Genotype T-DNA insertion in *tt19-8*
tt19-8_geno_RP	TATCCGAAATCTCTTCCCACC	
rdr6-11_geno_F	TACTGTCCCTGGCGATCTCT	Genotype mutation in *rdr6-11*
rdr6-11_geno_R	GGAACCTCAGTGTCAACCTCG	
rdr6-15_geno_LP	GGTTCTCCCTTTTTCGCATAC	Genotype T-DNA insertion in *rdr6-15*
rdr6-15_geno_RP	GCTGCAAAATAAGCACAAAGC	
sgs3-11_geno_F	CGTGTTAATGCATCTGTTATGT	Genotype mutation in *sgs3-11*
sgs3-11_geno_R	GATGAAGCTTGACACTTCCT	
sgs3-14_geno_LP	AAATTTGGAGTCCAGAATCGG	Genotype T-DNA insertion in *sgs3-14*
sgs3-14_geno_RP	CAAAGCATCGGAATCATTCTC	
dcl4-2e_geno_F	GCTAGAGCCACACATGAAATG	Genotype mutation in *dcl4-2e*
dcl4-2e_geno_R	TCATCATGTGGAAGCCTAGAAC	
dcl4-2t_geno_LP	TTTGCCAGTCTTACAAGTGGG	Genotype T-DNA insertion in *dcl4-2t*
dcl4-2t_geno__RP	GAGGCACCATATAGCAGCTTG	
tds4-4_geno_LP	CTGCTTTGAAAGAAGGCACAC	Genotype T-DNA insertion in *tds4-4*
tds4-4_geno_RP	AGCCGGAGAAGAGTTTTTCAG	
tt5-4_geno_LP	GTGGCTATATGGAAACAATTAGGG	Genotype T-DNA insertion in *tt5-4*
tt5-4_geno_RP	CTTTATTCTCCACTTGAGTACCGC	
tt19 S1/8_geno_F	CTGGTACAGAACTGCTGATGAT	Genotype mutation in tt19-8 S1/8
tt19 S1/8_geno_R	CCCATCGGTTAATATGCTCTTC	
tt19 S2_geno_F	GTCTTGGGTAATTGATTGCTTT	Genotype mutation in tt19-8 S2
tt19 S2_geno_R	TTAGAGACGCTGAGCAAGAA	
tt19 S4_geno_F	CTAGCATTCTCAGCCAATCAAC	Genotype mutation in tt19-8 S4
tt19 S4_geno_R	CTGACTGAAGACAACATCCC	
tt19 S5_geno_F	CGTGTTAATGCATCTGTTATGT	Genotype mutation in tt19-8 S5
tt19 S5_geno_R	GATGAAGCTTGACACTTCCT	
tt19 S7_geno_F	CTTATTTTTATGCACAAACTAGGTATGG	Genotype mutation in tt19-8 S7
tt19 S7_geno_R	GTTATCAAGTGAAATGAGATCATACC	
Actin2_F	TGCCAATCTACGAGGGTTTC	RT-qPCR for Actin2
Actin2_R	TTCTCGATGGAAGAGCTGGT	
PAP1_F	CGACTGCAACCATCTCAATG	RT-qPCR for PAP1
PAP1_R	TGTCCCCCTTTTCTGTTGTC	
GSTF5_F	TGACCAGAAGAAGCCGAGTT	RT-qPCR for GSTF5
GSTF5_R	CCCAGGTCAGTGTTGATGTG	
GSTF5-DT1-BsF	ATATATGGTCTCGATTGGTCCCAGTTTTCCTAGACGGTT	Constructs for two guide DNAs of GSTF5
GSTF5-DT1-F0	TGGTCCCAGTTTTCCTAGACGGTTTTAGAGCTAGAAATAGC	
GSTF5-DT2-R0	AACGTACATGGCTCGCGTTTATCAATCTCTTAGTCGACTCTAC	
GSTF5-DT2-BsR	ATTATTGGTCTCGAAACGTACATGGCTCGCGTTTATCAA	
GSTF5_CRISPR_F	ATGGGTTCCTCCATATTATTGGTTTTG	Genotype indels in GSTF5 CRISPR lines
GSTF5_CRISPR_R	ATGGACAGTCGCTATGTATTCAGATAT	

Supplementary references

1. Kitamura, S., Shikazono, N., Tanaka, A. *TRANSPARENT TESTA 1*9 is involved in the accumulation of both anthocyanins and proanthocyanidins in *Arabidopsis*. *Plant J.* **37**, 104-114 (2004).
2. Shikazono *et al*. Mutation rate and novel tt mutants of *Arabidopsis thaliana* induced by carbon ions. *Genetics* **163**, 1449-1455 (2003).
3. Li *et al*. The *Arabidopsis tt19-4* mutant differentially accumulates proanthocyanidin and anthocyanin through a 3' amino acid substitution in glutathione *S*-transferase. *Plant Cell Environ*. **34**, 374-388 (2011).
4. Sun *et al*. *Arabidopsis* TT19 functions as a carrier to transport anthocyanin from the cytosol to tonoplasts. *Mol Plant*. **5**, 387-400 (2012).
5. Wangwattana *et al*. Characterization of PAP1-upregulated glutathione *S*-transferase genes in *Arabidopsis thaliana*. *Plant Biotechnol*. **25**, 191-196 (2008).
6. Appelhagen *et al*. Update on *transparent testa* mutants from *Arabidopsis thaliana*: characterisation of new alleles from an isogenic collection. *Planta* **240**, 955-970 (2014).
