# Supplementary material for: Synergy between the anthocyanin and RDR6/SGS3/DCL4 siRNA pathways expose hidden features of Arabidopsis carbon metabolism
Source: Nat Commun. 2020 May 15;11:2456. doi: 10.1038/s41467-020-16289-3 (PMC7229025; doi:10.1038/s41467-020-16289-3)
Supplement: Supplementary file 3 — Reporting Summary [file 41467_2020_16289_MOESM3_ESM.pdf]

Corresponding author(s): Erich Grotewold
Last updated by author(s): Apr 20, 2020

Reporting Summary

Nature Research wishes to improve the reproducibility of the work that we publish. This form provides structure for consistency and transparency in reporting. For further information on Nature Research policies, see Authors & Referees and the Editorial Policy Checklist .

Statistics

For all statistical analyses, confirm that the following items are present in the figure legend, table legend, main text, or Methods section.

n/a	Confirmed	
☐	☒	The exact sample size (*n*) for each experimental group/condition, given as a discrete number and unit of measurement
☐	☒	A statement on whether measurements were taken from distinct samples or whether the same sample was measured repeatedly
☐	☒	The statistical test(s) used AND whether they are one- or two-sided<br>*Only common tests should be described solely by name; describe more complex techniques in the Methods section.*
☒	☐	A description of all covariates tested
☐	☒	A description of any assumptions or corrections, such as tests of normality and adjustment for multiple comparisons
☐	☒	A full description of the statistical parameters including central tendency (e.g. means) or other basic estimates (e.g. regression coefficient) AND variation (e.g. standard deviation) or associated estimates of uncertainty (e.g. confidence intervals)
☐	☒	For null hypothesis testing, the test statistic (e.g. *F*, *t*, *r*) with confidence intervals, effect sizes, degrees of freedom and *P* value noted<br>*Give P values as exact values whenever suitable.*
☒	☐	For Bayesian analysis, information on the choice of priors and Markov chain Monte Carlo settings
☒	☐	For hierarchical and complex designs, identification of the appropriate level for tests and full reporting of outcomes
☒	☐	Estimates of effect sizes (e.g. Cohen's *d*, Pearson's *r*), indicating how they were calculated

Our web collection on statistics for biologists contains articles on many of the points above.

Software and code

Policy information about availability of computer code

Data collection	Agilent ChemStation Rev. B. 02. 01-SR2, Waters MassLynx v4.1 SCN805, Thermo QuantStudio Real-Time PCR software v1.3.
Data analysis	Waters MassLynx v4.1 SCN805, Graphpad Prism 6.0c, ProteoWizard version 3, XCMS online, FastQC v0.11.5, Bowtie2 v2.3.4, Samtools v1.6, Bcftools v1.2, Vcftools v0.1.15, Hisat2 v2.1, FeatureCounts v1.5, R package DESeq2 v1.24, Mfuzz v2.44, Adapter Trimming for Small RNA Sequencing toll v0.3.2, Bowtie v1.1.2, edgeR v3.26.5

For manuscripts utilizing custom algorithms or software that are central to the research but not yet described in published literature, software must be made available to editors/reviewers. We strongly encourage code deposition in a community repository (e.g. GitHub). See the Nature Research guidelines for submitting code & software for further information.

Data

Policy information about availability of data

All manuscripts must include a data availability statement. This statement should provide the following information, where applicable:

- Accession codes, unique identifiers, or web links for publicly available datasets
- A list of figures that have associated raw data
- A description of any restrictions on data availability

The raw reads of RNA-seq and small RNA-seq were deposited in the NCBI Gene Expression Omnibus (GEO) and whole genome re-sequencing data were uploaded to Sequence Read Archive (SRA) under accession numbers GSE136680 [https://www.ncbi.nlm.nih.gov/geo/query/acc.cgi?acc=GSE136680], GSE136901 [https://www.ncbi.nlm.nih.gov/geo/query/acc.cgi?acc=GSE136901], and PRJNA562949 [https://www.ncbi.nlm.nih.gov/Traces/study/?acc=PRJNA562949], respectively. Source data underlying Figs 2, 3, and 5 and Supplementary Figs 6, 7, 9, 10b, c, e, 12a, b, and 13 are provided as a Source Data file.

Field-specific reporting

Please select the one below that is the best fit for your research. If you are not sure, read the appropriate sections before making your selection.

[x] Life sciences [ ] Behavioural & social sciences [ ] Ecological, evolutionary & environmental sciences

For a reference copy of the document with all sections, see nature.com/documents/nr-reporting-summary-flat.pdf

Life sciences study design

All studies must disclose on these points even when the disclosure is negative.

Sample size	For whole genome re-sequencing, sample sizes were 50 Arabidopsis plants pooled for extraction of genomic DNA following the reference "User guide for mapping-by-sequencing in Arabidopsis" (Fig.3, PMID: 23773572) that recommended a sample size of 50 as sufficient.<br>For anthocyanin inductive conditions (AIC), the sample size for each biological experimental replicate corresponded to ~100 Arabidopsis seedlings as determined to be sufficient by previous studies (PMID: 20085894 and 17921343).<br>For mutant segregation analysis, sample sizes were 150 Arabidopsis plants or more to analyze the phenotypes and provide robust segregation information as calculated to be be sufficient by previous studies (PMID: 6517052, 7343418, and https://doi.org/10.1007/BF00023206).
Data exclusions	No data was excluded.
Replication	At least three independent biological experiments were carried out and used for statistical analyses. The data from all replicates was included in the manuscript and all attempts at replication were successful.
Randomization	Plants were randomized in the same growth chamber from the same batch of seeds in different flats. The order and location of the samples was randomized during analysis.
Blinding	Blinding was not necessary for the analyses here carried out since control groups were included in all the experiments.

Reporting for specific materials, systems and methods

We require information from authors about some types of materials, experimental systems and methods used in many studies. Here, indicate whether each material, system or method listed is relevant to your study. If you are not sure if a list item applies to your research, read the appropriate section before selecting a response.

Materials & experimental systems

n/a	Involved in the study	
[x]	[ ]	Antibodies
[x]	[ ]	Eukaryotic cell lines
[x]	[ ]	Palaeontology
[x]	[ ]	Animals and other organisms
[x]	[ ]	Human research participants
[x]	[ ]	Clinical data

Methods

n/a	Involved in the study	
[x]	[ ]	ChIP-seq
[x]	[ ]	Flow cytometry
[x]	[ ]	MRI-based neuroimaging
