# Supplementary material for: Synergy between the anthocyanin and RDR6/SGS3/DCL4 siRNA pathways expose hidden features of Arabidopsis carbon metabolism
Source: Nat Commun. 2020 May 15;11:2456. doi: 10.1038/s41467-020-16289-3 (PMC7229025; doi:10.1038/s41467-020-16289-3)
Supplement: Supplementary file 4 — Description of Additional Supplementary Files [file 41467_2020_16289_MOESM4_ESM.pdf]

Description of Additional Supplementary Files

File name: Supplementary Data 1
Description: Summary of total aligned features between *tt19* suppressor lines or Col-0 and *tt19-8* by pairwise analyses using XCMS online.

File name: Supplementary Data 2
Description: List of 2,642 genes that were differential expressed between *tt19-8* and Col-0 under AIC.

File name: Supplementary Data 3
Description: Expression and Mfuzz clustering of 92 flavonoid-related genes in *tt19-8* and *tt19* suppressors against Col-0 under AIC.

File name: Supplementary Data 4
Description: Expression and Mfuzz clustering of 244 shared genes in all *tt19* suppressors against *tt19-8* under AIC.

File name: Supplementary Data 5
Description: Small RNA-seq differential expression analysis for miRs, tasiRNAs, and siRs among Col-0, *tt19-8*, *tt19-8* S2, and *tt19-8* S7 under AIC.

File name: Supplementary Data 6
Description: Prediction of siR6611 target sites by psRNAtarget analysis.
