# Supplementary material for: Synergy between the anthocyanin and RDR6/SGS3/DCL4 siRNA pathways expose hidden features of Arabidopsis carbon metabolism
Source: Nat Commun. 2020 May 15;11:2456. doi: 10.1038/s41467-020-16289-3 (PMC7229025; doi:10.1038/s41467-020-16289-3)
Supplement: Supplementary file 2 — Peer Review [file 41467_2020_16289_MOESM2_ESM.pdf]

Reviewers' comments:

Reviewer #1 (Remarks to the Author):

The manuscript by Jiang et al. presents a quite novel finding in the well-studied flavonoid system, uncovering a role for components of the siRNA pathway in controlling what is likely a localization mechanism for these plant-specific compounds. The study adds a lot of new information, raising many new questions that will indeed change thinking regarding the competing mechanisms that control flux/substantial carbon flow in specialized metabolism. The authors have done an excellent job of using the power of Arabidopsis, combining classical genetics with leading-edge genomic technologies, including whole-genome resequencing to identify mutations associated with reversion of the tt19 phenotype, extensive RNASeq analyses, comparison of metabolite levels LC-MS, and global analysis of small RNAs.

A few comments/suggestions:

The work appears to have been carefully carried out and the statistical analysis is solid, with a small comment for Figure 2 below.

Page 3: Some mention about the firm evidence for involvement of ABC transporters, including in Arabidopsis, should be included here.

Page 4, line 54 – the reduction in anthocyanin pigments is in what tissues.

Page 5, line 89 – there have been a number of previous reports of feedback control between flavonol and anthocyanin biosynthesis, e.g. Liu et al, PNAS 99: 14578-14583 (2002), a fact that might be mentioned.

Page 5, line 95 – the difference between a “push” and a “pull” needs to be explicitly described here.

The presentation of Figure 2 on page 9 is confusing, but crucial to the conclusion regarding function of the suppressors. With regard to the figure itself, it would be somewhat easier to follow if panel a were transcript levels and panel b the anthocyanin levels. It also be helpful to use a, b, c to indicate significant differences among the various genetic backgrounds. In the text, the authors should add a possible explanation for why tt19-8 PAP1-D seedlings had similar anthocyanin levels to tt19-8 seedlings, despite having higher PAP1 mRNA levels (line 196). Then something should be said about why the rdr6-11 line was included in this analysis; it would also not hurt to remind the reader that the S4 and S8 suppressors represent mutations in this gene. Then the next sentence (line 196) should be simplified to separate comparisons with the tt19-8 seedlings and the tt19-8 PAP1-D seedlings, something like, “Interesting, introducing the S4 or S8 mutations into the tt19-8 background resulted in an elevation of PAP1 mRNA and a small increase in anthocyanin levels relative to tt19-8. However, relative to tt19-8 PAP1-D seedlings, the tt19-8 S4 and S8 lines accumulated significantly higher levels of anthocyanins, despite lower PAP1 mRNA.”

Line 222, why was it expected that the tt19-8 seedlings would have an increase in flavonols/quercetin? Why is line 223, “However”?
age 11, line 228 – a bit more detail could be used here, including mentioning that quantification was by MRM and using authentic standards.

Pages 12 and 14 – unlike the convention for maize, genes encoding Arabidopsis flavonoid enzymes are typically referred to by the abbreviation of the enzyme name – so At5g13930 is CHS, not TT4; the same is true for TT5, TT7, TT3, TT13, TT10 in that section.

In general, the repeated use of “pathways” to refer to the two different control mechanisms/systems

is confusing, particularly when pathway is also used to refer to flavonol/anthocyanin/proanthocyanidin metabolism.

The discussion needs work. It is difficult to follow the logic of the conclusions as written, at least for me. First of all, the difference between a "push" and a "pull" needs to be defined better, as mentioned above. The text in this section is overall redundant, which does not help. The text in the paragraph on page 17 (lines 383-399) has a lot of repetition with what comes on page 18 and actually creates more confusion. There is one very nice statement (lines 410-413) that has a clarity that should be aimed for elsewhere in this section. A better use of paragraphs with defined topic sentences might help. In addition, the authors should refer to how this work relates to current thinking about GSTs and their role in stabilization/transport of flavonoids.

The manuscript would also benefit from some reworking of the text for better English usage and clarity, including consistent use of past tense for current results and present for those previously published. A pdf with a few suggestions is being provided.

Reviewer #2 (Remarks to the Author):

In this manuscript the authors identify suppressors of tt19 and very surprisingly, all are in the small RNA silencing pathway genes, RDR6/SGS3/DCL4. Supression leads to more anthocyanin and more flavonoids from the same general flavonol pathway. One idea was that this leads to more PAP1 by reducing degradation of the mRNA. However, they show that increasing PAP1 does not have the same effect.

They go on to show that genes in flavonoid pathway are affected by the suppressors and that this may (or appears to?) account for the differential amount of carbon shunted through the pathway in the suppressor mutants. In the absence of TT19 the levels of the flavonoids in tt19/suppressor double mutants go to higher levels 2X to 5X above tt19 alone.
In the single suppressor mutants, the total flavonoids go up and the ratio of flavonols to anthocyanins also goes up over slightly wt. In the tt19/suppressor doubles, the total amount of flavonoids goes up a lot but the ratio matches that of tt19, low anthocyanins higher flavonols.

I think the paper has a lot of positive qualities and will be of both general interest and interest to workers in the field for the following reasons:

• It employs a classical genetics screen that identifies some very curious suppressing loci, all components of the RNAi pathway. A nice example of "just when you thought we knew it all regarding regulation of the flavonoid pigment pathway...surprise, something new and unexpected".
• It revisits an old classic Arabidopsis mutant, tt19, an apparent GST. One could not have predicted the types of suppressors they found. More logical would have been upregulation of the biosynthetic pathway, or of another GST.
• The authors were thorough and logical with follow up experiments (like overexpressing that other GST, and similarly PAP1, to test different hypothesis as to how the suppressor mutations are working). Even if these hypotheses weren't validated, these were logical things to try and rule out. And they show that these types of suppressors probably would not show up in their screen.
• The flavonoid profiling was interesting, the finding/discussion of the "push vs pull" of carbon into the pathway in the suppressor lines, which to my knowledge is a novel interpretation of what happens when antho production is increased in some other way (like PAP1 overexpression).
• I was also intrigued that PAs were not rescued by the suppressor mutations, although they didn't say much about it besides making the observation.
• Overall, it is interesting and unanticipated the discovery that TT19 (a humble GST just thought to be influencing transport) could be doing so much more on a global scale regarding the regulation of

flavonoid profiles, pathway regulation, etc. Also surprising and novel is the connection between TT19 and the RNAi pathway, with one of the suppressor loci previously shown to regulate PAP1.
• To play devil's advocate, this paper raises way more questions than it answers via some interesting discoveries. There is quite a bit of confusion regarding the nature of the relationship between TT19 and the RNAi pathway, how mutations in different components of the RNAi pathway compensate for a lack of GST activity. But again, they do try to answer some logical questions, try to provide some mechanism, they just don't get any solid mechanistic answers. Otherwise, the science seems solid and they have tons of data.

The following are a few comments they may want to address:
• Fig 6. "Rather, what our studies indicate is that carbon is significantly "pushed" into the flavonoid pathway in the tt19 suppressor lines, a phenomenon that does not occur in the single mutants (tt19-8, rdr6-11, sgs3-11, or dcl4-2e; Fig. 6)." Doesn't fig 6 show the opposite of this? tt19 shows no total change but a big scew away from anthocyanins. rdr6-11, sgs3-11, and dcl4-2e all show a significant increase in total flavonoids (50 to 80%) and a slight scew away from Anthos (may not be significant but they all show this scew). Could it be that the absolute level of Anthos doesn't change much from wt, but the tt19/suppressors just show a big increase in flavonols. The question then becomes, why does tt19 enhance the increase in flavonols in the smallRNA pathway mutants?
• I suspect they may have looked at upstream mybs in rdr6 etc mutants? These are the mybs that regulate the early steps in anthocyanin/proantho, CHS, CHI,--. What happens to these in the suppressor mutants. It would seem to be important to upregulate these in order to send more C through the system. Can they disucss?
• It would be great to have a conceptual figure of some sort. The paper is very dense and detailed and will be difficult to read especially for people outside the field. The paper might be helped if even more of the data were moved to the supplemental and a model is presented. Even if it's one that shows where the next questions are.

Reviewer #3 (Remarks to the Author):

In the study described in the manuscript titled "Mutants in the RDR6/SGS3/DCL4 small RNA pathway expose hidden features of Arabidopsis metabolism", the research team find that mutations in the genes encoding RDR6, SGS3, and DCL4 could suppress the inability of transparent testa19 (tt19) mutant plants to produce anthocyanins. The group finds that these suppressors push carbon towards flavonoid biosynthesis in the absence of TT19 function, which allows the partial rescue of the anthocyanin deficient phenotype in these mutant plants. Thus, the group concludes there is synergistic regulation of carbon metabolism mediated by genetic/epigenetic mechanisms through controlling metabolic fluxes. The presented data is all high quality and supports the conclusions that can be drawn by the study and presented in the manuscript. However, there is a major and serious missing component in this study that really detracts from my enthusiasm and the overall general interest in this current study. Specifically, there is no evidence provided for the direct link between the mutations in the genes encoding RDR6, SGS3, and DCL4 and the effects on carbon metabolism. None of the current evidence provides any mechanistic understanding or even a correlative link between mutations in a small RNA producing pathway and differential carbon metabolism in the resulting mutant plants. Until a clear set of experiments to provide direct evidence between the synergistic regulation mediated by these two pathways in carbon flux this paper will not be of significant general interest, but will only be read and cited by a very specific and focused group of plant metabolic researchers. If the researchers can provide a clear mechanistic link between the RDR6 small RNA pathway and carbon metabolism in Arabidopsis then this study would be of more broad interest. However, without this evidence I cannot recommend publication of this study in its current form.

Addressing the Editor's Comment

Comment: Editorially, we think providing a clear set of experiments to link the RDR6 small RNA pathway to carbon metabolism (i.e. revealing the underlying mechanism) is the prerequisite for our further consideration (Reviewer #2 and #3).

Addressing Comment: We provide here three new sets of experiments that significantly contribute to revealing the underlying mechanism linking between flavonoid biosynthesis, the RDR6 small RNA pathway and carbon metabolism. I provide, as follows, a short synopsis of each, with the relevant new figures/datasets:

1) We show that enhanced carbohydrate metabolism provides the source of the additional carbon required for increased flavonoid accumulation (new Supplementary Figs. 8 and 9)
2) We demonstrate that a sucrose synthase (encoded by *SUS4*) is a target for a siRNA that requires both a TT19-controlled miRNA (miRNA161.2) and a RDR6-SGS3-DCL4 controlled siRNA (Supplementary Fig. 12 c, d)
3) We demonstrate that mutation of a second anthocyanin biosynthesis gene (*TDS4*) has a similar synergistic effect with the *rdr6* mutation on carbon flux to the flavonoid pathway (new Fig. 5).

As described below in response to the reviewers' comments, and more extensively described in the revised manuscript, these new findings permitted us to advance a model of how central and specialized metabolism might be coordinated through the RDR6-SGS3-DCL4 siRNA system.

Responses to the Reviewers' Comments:

Reviewer 1:

Comment 1: Page 3: Some mention about the firm evidence for involvement of ABC transporters, including in Arabidopsis, should be included here.

Response to Comment 1: The text has been modified with new references included as part of the following sentence: "Key players in this process are a group of conserved glutathione *S*-transferase (GST) proteins and some ABC transporters that interact with anthocyanins[15-17] and are believed to facilitate their transport across the tonoplast[18, 19]."

Comment 2: Page 4, line 54 – the reduction in anthocyanin pigments is in what tissues.

Response to Comment 2: The text has been modified to now read: "…the *tt19-1* mutant shows a ~90% reduction of anthocyanin pigments in seedlings and pale brown seed coats".

Comment 3: Page 5, line 89 – there have been a number of previous reports of feedback control between flavonol and anthocyanin biosynthesis, e.g. Liu et al, PNAS 99: 14578-14583 (2002), a fact that might be mentioned.

Response to Comment 3: Most of the studies (including the one mentioned above) relate to competition between the flavonol and anthocyanin branches of the Arabidopsis flavonoid pathway, and don't really relate to the issue of feedback control. Nevertheless, we added the following sentence to acknowledge these previous studies: "Previous studies already showed effects on other branches of the *Arabidopsis* flavonoid pathway when flux to flavonols or anthocyanins is perturbed[40-42]."

Comment 4: Page 5, line 95 – the difference between a "push" and a "pull" needs to be explicitly described here.

Response to Comment 4: We appreciate this comment as more clarity on what we refer to as 'push' and 'pull' in the context of this study was clearly warranted. Thus, we added a paragraph in the introduction and now we more carefully explain the context in which the two terms were used throughout the manuscript.

Comment 5: The presentation of Figure 2 on page 9 is confusing, but crucial to the conclusion regarding function of the suppressors. With regard to the figure itself, it would be somewhat easier to follow if panel a were transcript levels and panel b the anthocyanin levels. It also be helpful to use a, b, c to indicate significant differences among the various genetic backgrounds.

Response to Comment 5: Thanks to the reviewer for the comment. As recommended, Figure 2 has been modified as follows: Panel (a) shows now the *PAP1* mRNA levels and panel (b) shows now anthocyanin accumulation levels. We added to both panel letters representing the significant differences between the various lines, and explained their meanings in the figure legend.

Comment 6: In the text, the authors should add a possible explanation for why tt19-8 PAP1-D seedlings had similar anthocyanin levels to tt19-8 seedlings, despite having higher PAP1 mRNA levels (line 196). Then something should be said about why the rdr6-11 line was included in this analysis; it would also not hurt to remind the reader that the S4 and S8 suppressors represent mutations in this gene. Then the next sentence (line 196) should be simplified to separate comparisons with the tt19-8 seedlings and the tt19-8 PAP1-D seedlings, something like, "Interesting, introducing the S4 or S8 mutations into the tt19-8 background resulted in an elevation of PAP1 mRNA and a small increase in anthocyanin levels relative to tt19-8. However, relative to tt19-8 PAP1-D seedlings, the tt19-8 S4 and S8 lines accumulated significantly higher levels of anthocyanins, despite lower PAP1 mRNA."
Response to Comment 6: Thanks to the reviewer for the comment. The text has been modified as suggested and now reads: "*PAP1* mRNA levels were about five-fold higher in *PAP1-D* seedlings under AIC, compared to wild-type seedlings (Fig. 2a), and *PAP1-D* seedlings showed ~60% increase in anthocyanin accumulation when compared to Col-0 wild-type seedlings (Fig. 2b). Yet, although the *PAP1* mRNA steady-state level in *tt19-8 PAP1-D* seedlings was comparable to that of *PAP1-D* (compare the pink and orange bars in Fig. 2a), the *tt19-8 PAP1-D* line failed to accumulate more anthocyanins than the *tt19-8* seedlings (compare the pink and red bars in Fig. 2b). The *tt19* suppressor lines that have mutations in *RDR6* (S4 with RDR6^{G664R} or S8 with RDR6^{W299stop}) resulted in an elevation of *PAP1* mRNA levels and an increase in anthocyanin levels relative to *tt19-8*. However, compared to *tt19-8 PAP1-D* seedlings, the *tt19-8* S4 and S8 lines accumulated larger quantities of anthocyanins, despite lower *PAP1* mRNA levels (Fig. 2)."

Comment 7: Line 222, why was it expected that the tt19-8 seedlings would have an increase in flavonols/quercetin? Why is line 223, "However"?
Response to Comment 7: We have changed the text to now read: "Consistent with previous studies[16], our results showed that *tt19-8* seedlings had an approximately 25% increase in total flavonol content, primarily represented by quercetin, compared to wild-type.", and we replaced "However" by "Unexpectedly".

Comment 8: Page 11, line 228 – a bit more detail could be used here, including mentioning that quantification was by MRM and using authentic standards.
Response to Comment 8: Thank you for pushing us to be clearer. The text has been modified to now read: "To determine whether this increase in flavonol accumulation in the suppressor lines reflected an overall increase in other flavonoid classes, the samples were first subjected to acid hydrolysis and then the absolute amounts of other flavonoids, including the flavones apigenin and luteolin, the flavanones naringenin and eriodictyol, the flavanonols dihydrokaempferol (DHK) and dihydroquercetin (DHQ), the anthocyandins perlargonidin, cyanidin, and peonidin, and the flavan 3-ol epicatechin were measured using authentic standards and multiple reaction monitoring (MRM) (Fig. 3 and Supplementary Fig. 7)."

Comment 9: Pages 12 and 14 – unlike the convention for maize, genes encoding Arabidopsis flavonoid enzymes are typically referred to by the abbreviation of the enzyme name – so At5g13930 is CHS, not TT4; the same is true for TT5, TT7, TT3, TT13, TT10 in that section.
Response to Comment 9: Habits are difficult to change. The text was corrected accordingly.

Comment 10: In general, the repeated use of "pathways" to refer to the two different control mechanisms/systems is confusing, particularly when pathway is also used to refer to flavonol/anthocyanin/proanthocyanidin metabolism.
Response to Comment 10: We see the reviewer's point, thus we changed to "system" when we refer to RDR6-SGS3-DCL4, and kept pathway for flavonol/anthocyanin/proanthocyanidin metabolism. As an example of how it reads now, here is a passage from the Abstract that was modified accordingly: "This synergy between mutations in components of the RDR6-SGS3-DCL4 siRNA system and the anthocyanin pathway reveals genetic/epigenetic mechanisms to control metabolic fluxes."

Comment 11: The discussion needs work. It is difficult to follow the logic of the conclusions as written, at least for me. First of all, the difference between a "push" and a "pull" needs to be defined better, as mentioned above. The text in this section is overall redundant, which does not help. The text in the paragraph on page 17 (lines 383-399) has a lot of repetition with what comes on page 18 and actually

creates more confusion. There is one very nice statement (lines 410-413) that has a clarity that should be aimed for elsewhere in this section. A better use of paragraphs with defined topic sentences might help.
Response to Comment 11: We appreciate the comment. We re-wrote the discussion, avoiding repetition with the results, and also incorporating all the new results. We tried to separate concepts in different paragraphs whenever possible. We hope that the reviewer finds it now easier to read.

Comment 12: In addition, the authors should refer to how this work relates to current thinking about GSTs and their role in stabilization/transport of flavonoids.
Response to Comment 12: The study was clearly born with the objective of trying to better understand the role of GSTs in the stabilization/transport of flavonoids, and this is stated in the manuscript. However, the nature of the suppressors threw us in a completely different direction that has clearly less to do with anthocyanin stabilization/transport, and much more with how cells coordinate central and specialized carbon allocation. Thus, the contributions of this study to our thinking of how GSTs participate in anthocyanin stabilization/transport has really not changed much, with the exception of the demonstration that AtGSTF5 cannot compensate for the absence of TT19 (and this is well-described in the text). I am afraid that any additional discussion on the role of TT19 in the stabilization/transport of flavonoids will only be speculative and distract readers from the main findings. We'd be happy to reconsider if the reviewer/editor feel strongly about this.

Comment 13: The manuscript would also benefit from some reworking of the text for better English usage and clarity, including consistent use of past tense for current results and present for those previously published.
Response to Comment 13: Thanks to the reviewer for the suggestion. The manuscript has been checked and modified accordingly.

Comment 14: A pdf with a few suggestions is being provided.
Response to Comment 14: Thanks very much to the reviewer for the providing the edited pdf. It was quite useful, and the suggestions were incorporated in the text.

<u>Reviewer 2:</u>
Comment 1: There is quite a bit of confusion regarding the nature of the relationship between TT19 and the RNAi pathway, how mutations in different components of the RNAi pathway compensate for a lack of GST activity. But again, they do try to answer some logical questions, try to provide some mechanism, they just don't get any solid mechanistic answers.
Response to Comment 1: We appreciate the reviewer's concern. We believe that this new version provides several mechanistic answers (see above, "Addressing the Editor's Comment") that involved not only significant additional experimentation, but also a more comprehensive integration of the data available. The discussion was reorganized and re-written, and as consequence we hope that the confusion was eliminated.

Comment 2: Fig 6. "Rather, what our studies indicate is that carbon is significantly "pushed" into the flavonoid pathway in the tt19 suppressor lines, a phenomenon that does not occur in the single mutants (tt19-8, rdr6-11, sgs3-11, or dcl4-2e; Fig. 6)." Doesn't fig 6 show the opposite of this? tt19 shows no total change but a big scew away from anthocyanins. rdr6-11, sgs3-11, and dcl4-2e all show a significant increase in total flavonoids (50 to 80%) and a slight scew away from Anthos (may not be significant but they all show this scew). Could it be that the absolute level of Anthos doesn't change much from wt, but the tt19/suppressors just show a big increase in flavonols. The question then becomes, why does tt19 enhance the increase in flavonols in the smallRNA pathway mutants?
Response to Comment 2: We apologize to the reviewer for not having been clearer in the previous version. I think that the current version explains what the reviewer perceives as a contradiction between our data and our interpretation much better. Just in case, I will try to explain it here in a few responses to address the specific questions posed:

> <u>Comment:</u> *Fig 6. "Rather, what our studies indicate is that carbon is significantly "pushed" into the flavonoid pathway in the tt19 suppressor lines, a phenomenon that does not occur in the single mutants*

(tt19-8, rdr6-11, sgs3-11, or dcl4-2e; Fig. 6)." Doesn't fig 6 show the opposite of this? tt19 shows no total change but a big scew away from anthocyanins.

Answer: The effect of *tt19* in skewing flux from anthocyanins to flavonols was described before, and what the figure shows is that the overall flux into the flavonoid pathway (anthocyanins + flavonols + …) is similar in *tt19* as in wild type. What we said in the previous version (which continues to be the case in the current version, hopefully just clearer) is that "carbon is significantly pushed into the flavonoid pathway in the *tt19* suppressor lines", and this is what Fig. 6 shows (could it be that the reviewer is confusing tt19 and the tt19 suppressor lines indicated as *tt19-8* S8 for example?) – for example, in *tt19-8 S8*, 875±77 nmol g^{-1}DW of carbon are coming into the flavonoid pathway, compared to 205±29 nmol g^{-1}DW in the wild-type Col-0.

Comment: *rdr6-11, sgs3-11, and dcl4-2e all show a significant increase in total flavonoids (50 to 80%) and a slight scew away from Anthos (may not be significant but they all show this scew). Could it be that the absolute level of Anthos doesn't change much from wt, but the tt19/suppressors just show a big increase in flavonols.*

Answer: The effect of *rdr6-11, sgs3-11, and dcl4-2e* on the anthocyanin/flavonol ratio is indeed not significant. And yes, the *tt19* suppressors show a big increase in flavonols, and enough of an increase in anthocyanins for us to identify them as suppressors. We could debate as to why the increase in anthocyanins is not larger, and my argument would be that it has to do with whatever function TT19 normally has in stability/sequestration.

Comment: *The question then becomes, why does tt19 enhance the increase in flavonols in the smallRNA pathway mutants?*

Answer: And this is exactly the point of the study: how the observed increase in flavonoids (mainly flavonols since *tt19* can't accumulate much anthocyanins, even when *PAP1-D* is present) is related to the identified mutations in the RDR6-SGS3-DCL4 siRNA system.

Comment 3: I suspect they may have looked at upstream mybs in rdr6 etc mutants? These are the mybs that regulate the early steps in anthocyanin/proantho, CHS, CHI,--. What happens to these in the suppressor mutants. It would seem to be important to upregulate these in order to send more C through the system. Can they disucss?

Response to Comment 3: We analyzed our RNA-seq results for all possible changes in transcription factors or enzymes that could explain the increased flux into the pathway. This included of course all known flavonoid related genes (including transcription factors such as MYBs that regulate early genes) and we summarize the results in Supplementary Table 4. In general terms, we find no major changes that would explain the increased flux, and the exceptions are discussed in the manuscript. However, the results with *PAP1-D* suggest that the increased flux into the pathway is unlikely to be achieved by a 'pull' effect, and we provide evidence and discuss that it is indeed a 'push' what makes that additional carbon available.

Comment 4: It would be great to have a conceptual figure of some sort. The paper is very dense and detailed and will be difficult to read especially for people outside the field. The paper might be helped if even more of the data were moved to the supplemental and a model is presented. Even if it's one that shows where the next questions are.

Response to Comment 4: We apologize for the density of the study. We added one conceptual model to explain how the synergy may happen as Supplementary Fig. 12d. We have been back and forth with respect to presenting a graphical model at the end of the manuscript, and we settled in trying to be much clearer in the discussion. But I would be happy to provide a high-level conceptual model that summarizes the results, if the reviewer and editor feel that it is still needed.

Reviewer 3:

Comment: Specifically, there is no evidence provided for the direct link between the mutations in the genes encoding RDR6, SGS3, and DCL4 and the effects on carbon metabolism. None of the current evidence provides any mechanistic understanding or even a correlative link between mutations in a small RNA producing pathway and differential carbon metabolism in the resulting mutant plants. Until a clear set of experiments to provide direct evidence between the synergistic regulation mediated by these two pathways in carbon flux this paper will not be of significant general interest, but will only be read and cited by a very specific and focused group of plant metabolic researchers. If the researchers can provide a clear

mechanistic link between the RDR6 small RNA pathway and carbon metabolism in Arabidopsis then this study would be of more broad interest.

Response to Comment: We appreciate the reviewer's concerns. We believe that this revised version supplies at least one link between the mutations in the genes encoding RDR6, SGS3, and DCL4 and the effects on carbon metabolism, and this is extensively discussed in the manuscript, as well as above in the section "Addressing the Editor's Comment".

Reviewers' comments:

Reviewer #1 (Remarks to the Author):

Dr. Grotewold and colleagues have done an outstanding job responding to the comments of the three reviewers and the editor. This includes adding new experimental evidence addressing a key concern of the third reviewer to demonstrate a link with sugar metabolism. This additional evidence adds substantially to the impact of the manuscript, which presents a quite new and important insights into the poorly-understand, yet critical, issue of how plants are able to manage a very complex, and metabolically-costly, specialized biochemistry.

The authors have also thoughtfully addressed each of the detailed comments provided by myself and one other reviewer, improving the overall presentation of the study. This has also made it much easier to follow the description of and logic behind the interpretation of the "dense" data. This includes a clear presentation of the push/pull terminology in the introduction and discussion sections.

A minor detail - the authors should change "or" to "and" in line 487.

Reviewer #2 (Remarks to the Author):

The revised manuscript shows much progress in providing a mechanism for tying RDR6-SGS3-DCL4 to TT19. Linking SUS4 and miRNA161.2 provides this link. And they have strengthened the story by showing that the TDS4 anthocyanin mutant behaves like TT19 in this regard.

I do not have any remaining significant criticisms.

Reviewer #3 (Remarks to the Author):

The authors have addressed my major concerns. However, could the authors find a way to definitively prove that the lack of anthocyanin production in the various mutants is the cause of the change in carbon fluxes? This would definitely improve the general interest of this study.

<u>Responses to the Reviewers' Comments:</u>
<u>Reviewer 1:</u>
Comment 1: A minor detail - the authors should change "or" to "and" in line 487.
Response to Comment 1: Thanks to the reviewer for the comment. The text has been modified as suggested.

<u>Reviewer 3:</u>
Comment 1: However, could the authors find a way to definitively prove that the lack of anthocyanin production in the various mutants is the cause of the change in carbon fluxes?
Response to Comment 1:
We appreciate the reviewer's concern. We provide here a new set of experiments that contributes to dissecting the relationship between flavonoid accumulation under a disabled RDR6-SGS3-DCL4 siRNA system and carbon metabolism. In a nutshell, our results show that it is not the lack of anthocyanins, but rather a dysfunction of the anthocyanin pathway that synergizes with the disabled RDR6-SGS3-DCL4 siRNA system.

To reach this conclusion, we generated the *tt5-4 rdr6-15* double mutant which is blocked in the formation of all flavonoids. This mutant showed no enhanced sugar catabolism, providing compelling evidence that it is a flavonoid metabolism dysfunction (rather than no flavonoids) that causes the change in carbon fluxes, when in combination with the disabled RDR6-SGS3-DCL4 siRNA system. The power of the *tt5-4 rdr6-15* double mutant is that it allowed us to supplement it with different compounds, including flavonols, and then monitor the effect on sugar catabolism. Our results conclusively demonstrate that flavonols alone (i.e., in the absence of anthocyanins) are insufficient, and that most likely it is not the ratio of flavonols/anthocyanins that is responsible. The most parsimonious explanation is that an intermediate specific to the anthocyanin branch of the pathway present in both *tds4-4* and *tt19-8*, but absent in *tt5-4*, is responsible for the synergy with the RDR6-SGS3-DCL4 system to promote enhanced sugar catabolism. Identifying the intermediate (or perhaps a metabolic product of the intermediate) will require a sophisticated set of new experiments, as they are not taken-up easily in feeding experiments. We added a new paragraph to the end of the Results Section describing these new results.

We truly hope that you will find that new results address all the concerns previously raised by the editor and reviewers.

REVIEWERS' COMMENTS:

Reviewer #3 (Remarks to the Author):

All of my comments and concerns have been adequately addressed in the revised version of the manuscript. I thank the authors for adding the additional experiments to address my continued concerns after the first round of revision.
